# A cross-sectional study examining the reliability and invariance of the Depression, Anxiety, and Stress Scale–21 (DASS-21) across ten countries/regions

**DOI:** 10.7717/peerj.21745

**Published:** 2026-09-29

**Authors:** Xiu-Mei Chen, Yang Li, Iqbal Pramukti, Daniel Kwasi Ahorsu, Shikha Kukreti, Ji-Kang Chen, Wan Ying Gan, Kamolthip Ruckwongpatr, Rashmi Gupta, Cheng-Kuan Lin, Dang Tran, Pham le An, Mark D. Griffiths, I-Hua Chen, Chung-Ying Lin, Olga Malas

**Affiliations:** 1 School of Information Engineering, Shandong Youth University of Political Science, Jinan, Shandong, China; 2 School of Culture and Communication, Shandong Youth Universtiy of Political Science, Jinan, Shandong, China; 3 Department of Community Health Nursing, Faculty of Nursing, Universitas Padjadjaran, Sumedang, West Java, Indonesia; 4 Department of Special Education and Counselling, Education University of Hong Kong, Ting Kok, Hong Kong; 5 Department of Nursing, College of Medicine, National Cheng Kung University, Tainan, Taiwan; 6 Department of Social Work, Chinese University of Hong Kong, Shatin, New Territories, Hong Kong; 7 Department of Nutrition, Faculty of Medicine and Health Sciences, Universiti Putra Malaysia, Serdang, Selangor, Malaysia; 8 Institute of Allied Health Sciences, College of Medicine, National Cheng Kung University, Tainan, Taiwan; 9 Cognitive and Behavioural Neuroscience Laboratory, Department of Humanities and Social Sciences, Indian Institute of Technology Bombay, Mumbai, India; 10 Institute of Public Health, National Yang Ming Chiao Tung University, Taipei, Taiwan; 11 Department of Environmental and Occupational Health, University of Medicine and Pharmacy at Ho Chi Minh City, Ho Chi Minh City, Vietnam; 12 Center of Family Medicine, University of Medicine and Pharmacy at Ho Chi Minh City, Ho Chi Minh City, Vietnam; 13 Nottingham Institute of Psychology, Nottingham Trent University, Nottingham, United Kingdom; 14 Chinese Academy of Education Big Data, Qufu Normal University, Qufu, Shandong, China; 15 Faculty of Education, Qufu Normal University, Qufu, Shandong, China; 16 Biostatistics Consulting Center, National Cheng Kung University Hospital, College of Medicine, National Cheng Kung University, Tainan, Taiwan; 17 College of Nursing, Kaohsiung Medical University, Kaohsiung, Taiwan; 18 Department of Psychology, Sociology and Social Work Faculty of Education, Psychology and Social Work, University of Lleida, Lleida, Spain

**Keywords:** Depression, Anxiety, and Stress Scale-21, Factor structure, Exploratory structural equation modeling, Measurement invariance, University students

## Abstract

**Background:**

The Depression, Anxiety, and Stress Scale-21 (DASS-21) is widely used to assess psychological symptoms among university students. However, its factor structure remains inconsistent, and few studies have employed advanced modeling approaches such as exploratory structural equation modeling (ESEM), particularly in cross-cultural contexts.

**Objectives:**

The present study evaluated the factor structure of the DASS-21 by comparing five models: unidimensional, oblique three-factor, bifactor, three-factor ESEM, and bifactor ESEM. For the best-fitting model, internal consistency and measurement invariance across cultural, linguistic, and demographic groups were further examined.

**Methods:**

Using secondary data from an international mental health project, this cross-sectional study analyzed responses from 12,059 university students (*M* = 20.37 years, *SD* = 1.98; age range: 18–34; 36.6% men and 63.4% women) across ten distinct countries/regions: Mainland China, Ghana, Hong Kong, India, Indonesia, Malaysia, Spain, Taiwan, Thailand, and Vietnam.

**Results:**

The three-factor ESEM model showed the best fit across the ten countries/regions with acceptable fit indices, satisfactory internal consistency (McDonald’s *ω* > 0.70), and acceptable internal structural validity evidence. Measurement invariance testing supported configural and metric invariance across all ten countries/regions, whereas scalar invariance was not achieved, limiting direct cross-cultural comparisons. This pattern was also observed across the seven language groups. In contrast, scalar invariance was established across age, gender, data collection mode (online versus offline), and pandemic timing (onset versus post-COVID-19), further highlighting potential cultural and linguistic influences on DASS-21 responses.

**Conclusions:**

These findings support the DASS-21’s robust internal structure and reliability across diverse student populations, while emphasizing caution in cross-cultural comparisons.

## Introduction

Recent large-scale studies have documented a high prevalence of depression, anxiety, and stress among university students worldwide (Healthy Minds Network, 2022; Paiva et al., 2025). A survey conducted by Healthy Minds Network (2022) reported that 44% of students experienced depressive symptoms, 37% reported anxiety symptoms, and 11% met trauma and stress-related disorders. Similarly, an umbrella review found pooled prevalence estimates of 35.4% for mild depression and 13.4% for severe depression, 40.2% for mild anxiety and 16.8% for severe anxiety, and 36.3% for stress (Paiva et al., 2025). These high rates of poor mental health symptoms among university students have been consistently associated with adverse outcomes, including reduced academic engagement, impaired career development, and lower life satisfaction (Hinkelman & Luzzo, 2007; Liu & Wang, 2024; Sinval et al., 2025).

Given that university years represent a critical window for prevention and early intervention (Duffy, 2023), the availability of reliable and valid screening tools is essential. Among these tools, the Depression, Anxiety, and Stress Scale-21 (DASS-21) has emerged as one of the most widely used instruments for assessing these symptoms in student populations (Bibi et al., 2020; Zanon et al., 2021). However, despite its widespread application, the factor structure of the DASS-21 remains inconsistent across studies, particularly in culturally diverse contexts (Yeung, Yuliawati & Cheung, 2020).

One potential reason for this inconsistency is methodological. Most existing research has relied on traditional confirmatory factor analysis (CFA) (Yeung, Yuliawati & Cheung, 2020; Dwight et al., 2024), which may not adequately capture the complexity and potential cross-loadings inherent in psychological constructs. To address this limitation, more flexible modeling approaches have been proposed. One such approach is exploratory structural equation modeling (ESEM), which integrates the strengths of both CFA and exploratory factor analysis (EFA). Despite its methodological advantages, ESEM remains underutilized in cross-cultural validation studies of the DASS-21, particularly in large-scale, multi-regional samples.

In addition to the factor structure of the DASS-21, measurement invariance is equally critical in cross-cultural validation studies. Without measurement invariance, any observed cross-cultural differences in DASS-21 scores could reflect measurement artifacts rather than true variations in depression, anxiety, or stress. In fact, measurement invariance has been largely neglected in contemporary psychological research. A systematic review by Maassen et al. (2025) reported that only 4% of the 929 comparisons examined had tested for measurement invariance, and such analyses were often poorly reported.

To advance these fields, the present study systematically evaluated five competing models: three CFA-based (unidimensional, oblique three-factor, bifactor) and two ESEM-based (ESEM, bifactor ESEM). The study aimed to identify the best-fitting structure and examine measurement invariance across university populations from ten distinct countries/regions: Mainland China, Ghana, Hong Kong, India, Indonesia, Malaysia, Spain, Taiwan, Thailand, and Vietnam. These countries/regions encompass markedly diverse cultural backgrounds, spanning both collectivist and individualistic orientations as well as diverse religious traditions, thereby providing a rigorous and comprehensive basis for evaluating the cross-cultural validity of the DASS-21.

Among the ten countries/regions, nine represent collectivist contexts, whereas Spain reflects a relatively more individualistic context. Prior research suggests that collectivist cultures tend to emphasize emotional restraint and somatic expressions of distress, whereas more individualistic cultures tend to endorse explicit psychological labeling of emotional difficulties (Ryder, Yang & Heine, 2002; Ryder et al., 2008). Consistent with this, Spain exhibits a relatively more individualistic cultural orientation, in which open emotional expression and the psychologization of distress are likely more common.

Despite sharing a collectivist orientation, the nine other countries/regions exhibit substantial cultural diversity, with religious and philosophical traditions further shaping how distress is expressed and interpreted. Mainland China, Hong Kong, Taiwan, and Vietnam are influenced by Confucianism, which encourages emotional restraint and self-regulation (Li, 2023). Malaysia and Indonesia, shaped by Islamic cultural values, often interpret psychological symptoms through a religious lens (Tanaka et al., 2025). Thai culture, rooted in Buddhist principles, particularly the ideal of inner peace, tends to minimize overt emotional fluctuations (Pholphirul & Srijamdee, 2025). Ghana’s cultural core is the Ubuntu philosophy, which emphasizes relationality, collective responsibility, and communal accountability (Udah et al., 2025), and may lead individuals to suppress personal distress in favor of group harmony. Similarly, Indian culture emphasizes social harmony and the acceptance by individuals of their circumstances based on karma (Chaturvedi, 2015), which may discourage direct emotional disclosure. Such cultural variations in emotional expression and symptom interpretation likely affect responses to the DASS-21, underscoring the need to critically evaluate its factor structure and measurement invariance across these countries/regions.

### Competing factor structure of the DASS-21

The DASS was developed by Lovibond & Lovibond (1995a) based on Clark & Watson’s (1991) tripartite model, which distinguishes a shared component of negative affect and unique features of depression (anhedonia) and anxiety (physiological hyperarousal). During its development, a third factor, stress, was empirically identified, characterized by symptoms such as nervous tension and irritability. This led to the establishment of a three-factor structure assessing symptoms of depression, anxiety, and stress.

Traditionally, three structural models of the DASS-21 have been tested using CFA: (1) the unidimensional model, which assumes that all items reflect a single general distress factor; (2) the oblique three-factor model, which posits three correlated but distinct constructs, namely depression, anxiety, and stress; and (3) the bifactor model, which allows each item to load on both a general distress factor and its corresponding specific factor, thus separating shared and unique sources of variance (Alfonsson, Wallin & Maathz, 2017). However, CFA adheres to the independent clusters model, which restricts each item to a single factor and discourages cross-loading estimation (Marsh & Alamer, 2024). This limitation can result in poor model fit and inflated inter-factor correlations (Marsh et al., 2014; Morin, 2023), potentially misrepresenting the true structure of psychological constructs (Alamer & Marsh, 2022). To address these limitations, ESEM, developed by Asparouhov & Muthén (2009), combines the flexibility of EFA (allowing cross-loadings) with the statistical rigor of CFA (*e.g.*, fit indices). This hybrid approach provides a more nuanced and accurate representation of the interrelationships among multidimensional constructs (Marsh et al., 2011; Sánchez-Carracedo et al., 2012).

Within the bifactor framework, the advantage of ESEM becomes even more critical. Unlike the bifactor CFA model, which still restricts cross-loadings among specific factors, the bifactor ESEM model allows such cross-loadings (Asparouhov & Muthén, 2009). This is particularly important for the DASS-21, given the consistently high intercorrelations among its depression, anxiety, and stress subscales (Mellor et al., 2014; Zhang et al., 2024). By permitting cross-loadings, the bifactor ESEM model better separates general distress from factor-specific variance, thereby providing a less biased estimate of each subscale’s unique contribution. Consequently, ESEM-based models, particularly the three-factor ESEM and bifactor ESEM, have been increasingly employed to validate the DASS-21 in diverse populations (Yılmaz Koğar & Koğar, 2022; Zhou et al., 2024).

Existing findings regarding the factor structure of the DASS-21 are inconsistent across studies and cultural contexts (Dwight et al., 2024; Yeung, Yuliawati & Cheung, 2020). For instance, the unidimensional model has been supported among Australian youth (Patrick, Dyck & Bramston, 2010) and Chinese medical staff (Jiang et al., 2022). The three-factor model showed better fit among adults (Gomez, Stavropoulos & Griffiths, 2020) and Filipino university students (Aruta et al., 2024). The bifactor model has been validated in cross-national university samples (Zanon et al., 2021; Zanon et al., 2025). The three-factor ESEM model was optimal among Italian, Chinese, and Spanish samples (Iannattone et al., 2024; Zhou et al., 2024). The bifactor ESEM model was supported in Serbian and Turkish populations (Jovanović, Gavrilov-Jerković & Lazić, 2021; Yılmaz Koğar & Koğar, 2022). Additionally, a four-factor structure has been proposed in a nonclinical sample, where anxiety items formed a distinct “panic” dimension (Alvarenga et al., 2024).

### Measurement invariance considerations for the DASS-21

The inconsistent factor structures of the DASS-21 observed across cultural groups reflect not only theoretical and structural complexities but also highlight a critical psychometric concern: measurement invariance. Beyond identifying variations in how symptoms cluster across cultures, it is essential to determine whether the scale functions equivalently across diverse populations. As previously mentioned, cultural norms may shape how individuals experience, label, and report emotional distress; some cultures may emphasize somatic symptoms, while others may encourage or discourage overt emotional expression (Ryder, Yang & Heine, 2002; Ryder et al., 2008).

Building on these cultural insights, empirical investigations into the DASS-21’s measurement invariance have produced mixed results. Examining the unidimensional model, item invariance was established across samples from Germany, Ghana, India, and New Zealand using the Rasch model (Adu et al., 2025). Examining the oblique three-factor model, configural invariance has been supported across six Asian countries (Oei et al., 2013), while scalar invariance has been observed between specific country pairs among adolescent samples from Australia, Mainland China, Chile, and Malaysia (Mellor et al., 2014), as well as among university students from Honduras and Colombia (Castillo-Díaz & Periañez, 2025). Similar evidence of scalar invariance has been reported among adult samples from the UK, USA, Russia, and Poland (Scholten et al., 2017). Furthermore, partial metric and scalar invariance have been identified between Pakistani and German university students (Bibi et al., 2020). Examining the bifactor model, configural invariance was supported across all eight countries, and partial scalar invariance was established in a subset of the countries examined by Zanon et al. (2021). In their subsequent work, multigroup CFA and the alignment method were used to further evaluate the measurement invariance of the DASS-21 across nine countries/regions. The findings indicated that scalar invariance for the oblique three-factor model across all nine countries/regions, and for the bifactor model across seven countries/regions (Zanon et al., 2025). Dispite these findings, no studies to date have tested measurement invariance using the ESEM framework, leaving a critical gap in integrating flexible structural models with cross-cultural validation.

Although university students may share similar academic stressors, cultural norms and response styles can still shape how symptoms are reported. Moreover, evidence for scalar invariance remains highly dependent on the population studied. These findings highlight the need for further research to examine measurement invariance using appropriate models, such as ESEM and bifactor ESEM, across culturally diverse university samples.

### The present study

While existing research has contributed substantially to the psychometric evaluation of the DASS-21, notable gaps remain. Few studies have incorporated advanced modeling techniques such as ESEM, and even fewer have examined measurement invariance across cultures using models that adequately accommodate the cross-loadings commonly observed among depression, anxiety, and stress items. To address these gaps, the present study investigated three interrelated research questions. First, we sought to determine which factor structure best represents the DASS-21 across culturally diverse university populations by comparing five competing models: unidimensional, oblique three-factor, bifactor, three-factor ESEM, and bifactor ESEM. Second, we examined whether the optimal model demonstrates adequate psychometric properties, including internal consistency. Finally, we tested whether the DASS-21 achieves measurement invariance across ten countries/regions: Mainland China, Ghana, Hong Kong, India, Indonesia, Malaysia, Spain, Taiwan, Thailand, and Vietnam, by evaluating configural, metric, and scalar invariance. These questions collectively address both the structural validity and cross-cultural applicability of the DASS-21 in university settings.

## Methods

### Research design

The present cross-sectional study analyzed secondary data from a large international mental health project led by the present research team. Data were collected between 2021 and 2023 across ten countries/regions: Mainland China, Ghana, Hong Kong, Indonesia, India, Malaysia, Spain, Taiwan, Thailand, and Vietnam (see Table S1). For the present study, only responses to the 21 items of the DASS-21 were extracted, as it was the only instrument consistently administered across all participating countries/regions. Although additional psychological measures were included in some countries/regions, these instruments varied and were not uniformly available.

The study procedure adhered to the Declaration of Helsinki, with ethical approval obtained from the respective research institutions’ ethics committees. The specific details are as follows:

Mainland China: Jiangxi Psychological Consultant Association/IRB ref: JXSXL-2022-Jul13; Ghana: Kwame Nkrumah University of Science and Technology/IRB ref: CHRPE/AP/612/23; Hong Kong: Survey and Behavioural Research Committee at the Chinese University of Hong Kong/IRB ref: SBRE-22-0186; Indonesia: Health Research Ethics Commission, Faculty of Nursing, Universitas Airlangga/ref: 2318-KEPK; India: Indian Institute of Technology Bombay Review Board/IRB ref: 2022/045; Malaysia: Jawatankuasa Etika Universiti Penyelidikan Melibatkan Manusia,/IRB ref: JKEUPM-2022-268; Taiwan: Institutional Review Board in the Chi Mei Medical Center, IRB ref: 11007006; Thailand: Human Research Ethics of National Cheng Kung University and endorsed by the ethics committee at Mahidol University, IRB ref: NCKU HREC-E-110-486-2; Vietnam: Board of Ethics in Biomedical Research in the University of Medicine and Pharmacy at Ho Chi Minh City, IRB ref: 1080/UMP-BOARD; Spain: It was considered exempt by the IRB of its institution (CEIM HUAV).

### Participants

Participants were university students recruited through convenience and snowball sampling with support from student affairs offices at each participating institution. More specifically, these offices first disseminated recruitment announcements to engage students from diverse faculties and across academic years. Participants then had the opportunity to invite their peers, thereby extending the samples through snowball sampling. All participants were assured of anonymity and informed of their right to withdraw at any time. Written informed consent was obtained from each individual. Participation was entirely voluntary, with no incentives provided. Due to the use of convenience and snowball sampling methods, the sample was not proportionally balanced across countries/regions. Notably, participants from Mainland China accounted for more than half of the total analytic sample (see Table S1).

The inclusion criteria comprised: (i) current enrollment as a university student and (ii) absence of previously self-reported diagnosed mental health disorders. As part of the sociodemographic questionnaire, participants responded to a single self-report item regarding any prior diagnosis of a mental health disorder. This item was used solely to determine eligibility based on the inclusion criteria and was not intended as a clinical screening or diagnostic assessment.

To verify that the recruited sample was sufficient for the planned analyses, sample size adequacy was evaluated through an a priori power analysis. For factor structure analysis using CFA and ESEM, the analysis was conducted with the *semPower* package in R (R Core Team, 2025). Based on three correlated factors (loadings = 0.70, correlations = 0.70), an alpha of 0.05, and testing the Root Mean Square Error of Approximation (RMSEA) ≤ 0.05 (close fit) against RMSEA = 0.08 (misfit), the required sample sizes were estimated as follows. For the oblique three-factor model (*df* = 186), 150–200 participants were required for 80% power and 200–250 for 90% power. For the three-factor ESEM model (*df* = 150, accounting for cross-loadings), 250–300 participants were required for both 80% and 90% power. These estimates, consistent with prior studies (Bibi et al., 2020; Gomez, Stavropoulos & Griffiths, 2020), support the adequacy of the present sample because each country/region contributed more than 400 participants, ensuring robust and stable parameter estimation.

### Measures

#### Demographic information form

Age and gender were collected *via* self-report as basic demographic variables. In addition, participants completed the DASS-21 in their native languages, using corresponding validated language versions: simplified Chinese (Zuo & Chang, 2008), Traditional Chinese (Moussa, Lovibond & Laube, 2001), English (Lovibond & Lovibond, 1995b), Bahasa Indonesian (Ifdil et al., 2022), Spanish (Patricia et al., 2002), Thai (NCHECR Thai translation, 2007), and Vietnamese (Tran, Tran & Fisher, 2013). A detailed list of language versions used in each region is provided in Table S1. No additional translation, cultural adaptation, or linguistic harmonization procedures were conducted within the present study.

#### DASS-21

The DASS-21 was used to assess the presence and severity of symptoms of depression, anxiety and stress among participants. The content of the three subscales is described below based on Lovibond & Lovibond (1995b) and other scholars (Şahin, Topkaya & Gençoğlu, 2022). The depression subscale assesses symptoms associated with anhedonia, dysphoria, inertia, feelings of worthlessness, lack of meaning in life, and reduced engagement in daily activities. The anxiety subscale assesses symptoms related to anxious affect, situational anxiety, autonomic arousal, and skeletal muscle effects. The stress subscale assesses symptoms related to nervous arousal, agitation, irritability, overreaction, impatience, and difficulty relaxing.

Each subscale of DASS-21 comprises seven items, with example items including *“I couldn’t seem to experience any positive feeling at all”* (depression), *“I was aware of dryness of my mouth”* (anxiety), and *“I found it hard to wind down”* (stress). Each item is rated on a four-point Likert scale ranging from 0 to 3, with total subscale scores ranging from 0 to 21. Higher scores indicate greater symptom severity.

### Procedures

Data collection methods varied depending on local infrastructure and conditions. Some countries/regions used country-specific online platforms, whereas others employed paper-and-pencil formats. Detailed information on data collection modes and study locations is provided in Table S1. To minimize temporal bias, data collection was aligned across countries/regions and conducted between June 2021 and October 2023 (also see Table S1 for timeline details).

### Statistical analyses

Prior to formal data analysis, data cleaning procedures were implemented. Responses with values outside the valid range (0–3) on any DASS-21 item were excluded. For online surveys, responses with a total completion time of less than 120 s were also excluded. This cutoff was based on the total length of the original survey (approximately 60 items) and guided by recommendations that response times below 2 s per item may indicate insufficient effort responding (Bowling et al., 2023). The 120-second threshold therefore reflected the overall survey length rather than the DASS-21 alone and was applied uniformly across all online platforms and countries/regions. The subsequent analyses comprised descriptive statistics, factorial validity testing, and measurement invariance assessment.

### Descriptive statistics

Descriptive statistics, including means, standard deviations, skewness, and kurtosis, were calculated for each item across the ten countries/regions, both for the full sample and country/region-specific subsamples. Multivariate normality was examined using Mardia’s test. If multivariate normality was not supported, weighted least squares mean and variance adjusted (WLSMV) estimation was applied for factorial validity testing and measurement invariance assessment; otherwise, maximum likelihood (ML) estimation was used.

### Factorial validity testing

Factorial validity of the DASS-21 was examined beginning with a one-factor baseline model. Following guidelines of ESEM methodology (Alamer & Marsh, 2022; Alamer & Alrabai, 2024; Swami, Maïano & Morin, 2023), model comparisons were conducted sequentially. The oblique three-factor model (representing the original DASS-21 structure) was compared with the three-factor ESEM model (allowing item cross-loading) using oblique Geomin rotation (epsilon = 0.5) (Asparouhov & Muthén, 2009). If both models demonstrated adequate fit, bifactor models, including bifactor CFA and bifactor ESEM, were further evaluated. If the three-factor ESEM model showed superior fit, only the bifactor ESEM model was retained for further analysis. To examine the robustness of findings, sensitivity analyses were conducted using alternative suitable estimators alongside the one employed for the main analyses.

Model fit was evaluated using multiple indices, including the chi-square statistic (*χ*^2^), the Comparative Fit Index (CFI), the Tucker-Lewis Index (TLI), RMSEA, and Standardized Root Mean Square Residual (SRMR). Given the sensitivity of *χ*^2^ to large sample sizes, greater emphasis was placed on CFI, TLI, RMSEA and SRMR, with CFI, TLI > 0.90, and RMSEA < 0.10, SRMR < 0.08 considered acceptable (Browne & Cudeck, 1992; Kline, 2023). To avoid overestimating fit in complex models with more parameters (*e.g.*, ESEM and bifactor ESEM), stricter criteria were applied: improvements were considered meaningful if ΔCFI > 0.010, ΔRMSEA > 0.015 and ΔSRMR > 0.015 (Kline, 2023).

Additionally, inter-factor correlations in the three-factor ESEM model were examined to assess the plausibility and differentiation of the factor structure, following recommendations by Marsh et al. (2014). Within the ESEM framework, examination of inter-factor correlations serves as a descriptive structural check of factor differentiation, without relying on predefined cutoff values or formal tests of discriminant validity. Internal consistency was evaluated using McDonald’s omega for each subscale across all ten countries/regions. The omega coefficient accounts for variance from multidimensional sources, making it a suitable reliability estimate (Cousi, Igier & Quintard, 2021), with values above 0.70 indicating acceptable internal consistency (Fornell & Larcker, 1981).

### Measurement invariance assessment

To enable valid comparisons across the ten countries/regions, a two-stage multi-group CFA approach was adopted. Due to substantial sample imbalance, in which Mainland China accounted for more than half of the total sample, a single multi-group model including all ten countries/regions failed to converge. To address this issue, a stratified subsampling strategy was employed to achieve comparable sample sizes across all ten countries/regions. More specifically, a random subsample of 15% of the Mainland China sample was drawn to match the sample sizes of the other countries/regions, and this procedure was repeated five times. Measurement invariance testing was then performed using each of these five 15% subsamples from Mainland China along with the full samples from the other nine countries/regions.

To account for potential confounding factors, supplementary invariance tests were conducted across language version (seven versions), age, gender, data collection mode (online *vs.* offline), and pandemic timing (onset *vs.* post-COVID-19) based on the World Health Organization’s official declaration (World Health Organization, 2023). Age groups were split at 20 years, reflecting typical university progression. Measurement invariance was tested through sequential model comparisons examining three levels: configural invariance (identical factor structure), metric invariance (equal factor loadings), and scalar invariance (equal intercepts). Model fit changes were evaluated using Chen’s criteria (Chen, 2007): ΔCFI > −0.01, ΔRMSEA < 0.015, and ΔSRMR < 0.03 for metric invariance, and ΔSRMR < 0.01 for scalar invariance.

## Results

### Data cleaning

The initial dataset comprised 12,348 university students. Data entries were excluded if they contained invalid DASS-21 responses (*i.e.,* values outside the 0-3 range) or, if the recorded response time was less than 120 s, suggesting inattentive responding. The participant selection process is illustrated in Fig. 1. The overall proportion of missing data was minimal (0.1%), with missing values limited to Item 11 (*n* = 1), Item 12 (*n* = 2), Item 14 (*n* = 2), Item 18 (*n* = 3), and age (*n* = 5). After data screening, the final analytic sample included 12,059 participants from ten countries/regions. The average age was 20.37 years (*SD* = 1.98; range = 18–34), with 4,414 men (36.6%) and 7,645 women (63.4%). Notably, slightly over half of the participants were from Mainland China (*n* = 6,278, 52.06%). A detailed breakdown by region is provided in Table S1.

**Figure 1:**
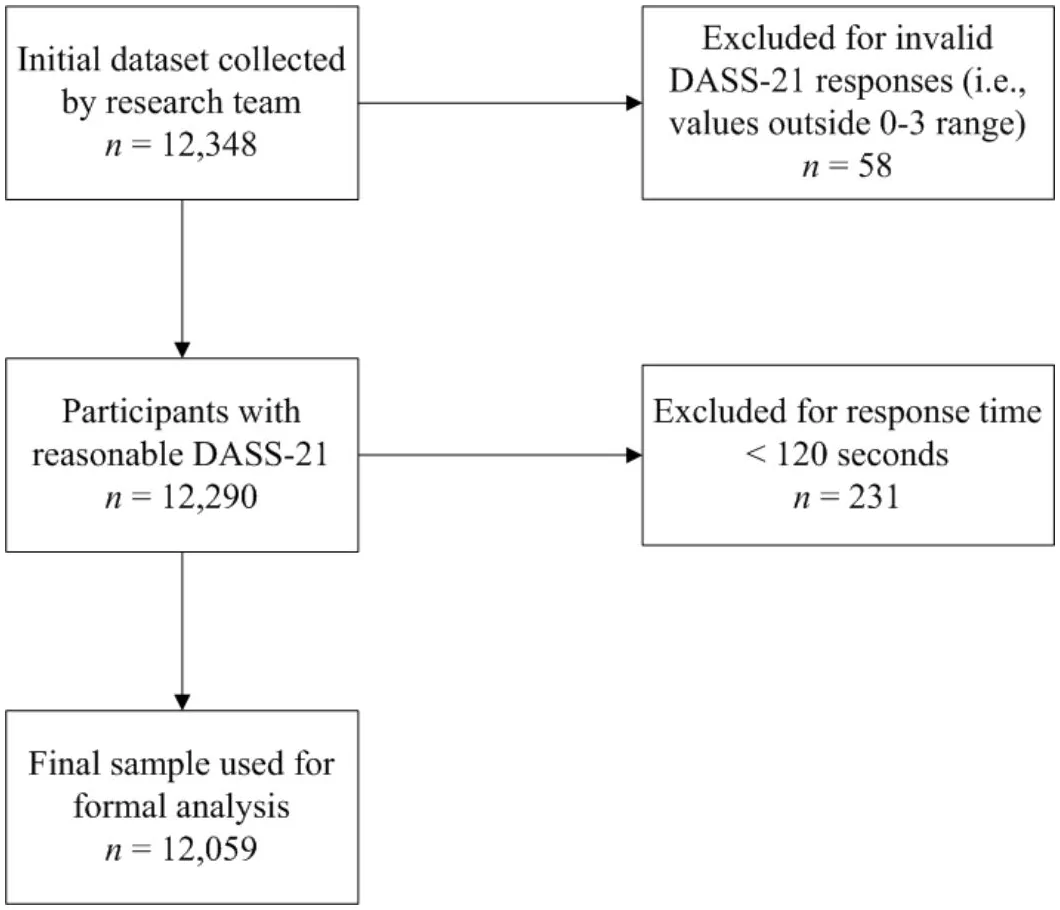
Flow diagram of participant selection.

### Descriptive statistics

Tables 1 to 3 present the item statistics including means (*SD*), skewness, and kurtosis for the full sample as well as stratified by the ten countries/regions. Most regional samples exhibited positive skewness and kurtosis values, with the notable exception of the Spanish sample, which showed predominantly negative values. Mardia’s multivariate normality test was significant for each country/region and the full sample (skewness = 60,007.56, kurtosis = 535.03), with skewness ranging from 4,777.56 (Spain) to 11,340.49 (Taiwan) and kurtosis ranging from 47.12 (Spain) to 460.10 (Mainland China), all *p* < 0.001. These results indicate clear violations of multivariate normality. Accordingly, WLSMV estimation was applied for the factorial validity and measurement invariance analyses, which is also well suited to the ordinal Likert-type response format of the DASS-21 items. Minor missing responses were addressed using full information maximum likelihood (FIML) estimation.

### Factor structure

Given the dataset imbalance, with participants from Mainland China comprising 52.06% of the total sample, pooled fit indices may be disproportionately influenced by the Mainland China subsample, potentially producing fit estimates that reflect the structure of this single subsample rather than the overall cross-national fit. To address this potential bias, model fit was therefore evaluated through country/region-specific analyses. When examined separately by country/region, the unidimensional structure demonstrated inadequate fit across all ten samples, with TLI values ranging from 0.749 (Indonesia) to 0.898 (Ghana and Taiwan) (see Table 4).

Subsequently, the oblique three-factor structure with the three-factor ESEM model was compared. The oblique three-factor model showed inadequate fit in the majority of countries/regions. Notable exceptions were observed in Ghana (CFI = 0.913, TLI = 0.902, RMSEA = 0.088, SRMR = 0.038) and Spain (CFI = 0.931, TLI = 0.923, RMSEA = 0.085, SRMR = 0.033). In contrast, the three-factor ESEM model exhibited ideal factorial validity across all ten countries/regions, with CFI and TLI values exceeding 0.95, RMSEA values ranging from 0.057 to 0.084, and SRMR values ranging from 0.016 to 0.038 (see Table 4).

Given that the oblique three-factor model did not demonstrate adequate fit, whereas the three-factor ESEM model showed acceptable model fit across all countries/regions, and following the guidelines established in previous research (Alamer & Marsh, 2022; Alamer & Alrabai, 2024; Swami, Maïano & Morin, 2023), a bifactor CFA model under these conditions was not considered. Instead, only a bifactor ESEM model was estimated. The bifactor ESEM model exhibited acceptable fit across all ten samples, with CFI and TLI values exceeding 0.95, RMSEA values ranging from 0.045 to 0.059, and SRMR values ranging from 0.014 to 0.029 (see Table 4).

However, the bifactor ESEM model did not yield improvements in model fit over the three-factor ESEM model that exceeded the predetermined thresholds, suggesting no substantial advantage. More specifically, all ΔCFI values comparing the bifactor ESEM model with the three-factor ESEM model were below the 0.01 cutoff (see Table 4). Therefore, the three-factor ESEM model was identified as the most appropriate structure for the DASS-21 across university students in all ten countries/regions. To examine the robustness of the findings against the choice of estimator, sensitivity analyses were conducted using maximum likelihood with robust standard errors (MLR) estimation. The results were consistent with those obtained from the WLSMV-based analyses. The model fit indices are presented in Table S2.

For the three-factor ESEM model, standardized factor loadings for all ten countries/regions are presented in Table S3. The results showed that primary factor loadings were positive and within a plausible range (0.03–0.90). No Heywood cases were detected because all factor loadings were below 0.95 and no negative primary loadings were observed. Although some negative loadings were present, these were limited to cross-loadings of items on non-target factors and were small in magnitude (ranging from −0.17 to −0.01). All primary factor loadings were statistically significant (*p* < 0.001), and the majority exceeded 0.30 across most countries/regions. Two exceptions were noted: Item 5 from the depression subscale and Item 9 from anxiety subscale exhibited primary factor loadings below 0.30 in more than half of the ten samples. Taken together, these findings further supported the three-factor ESEM as the best-fitting structure in the present study.

Following the identification of the three-factor ESEM model as the most appropriate structure, further analyses were conducted to examine its factor loadings, inter-factor correlations, and internal consistency. Notably, indices such as average variance extracted (AVE) and the heterotrait-monotrait ratio (HTMT) are not readily interpretable within the ESEM framework because the presence of cross-loadings complicates their computation and interpretation. Therefore, the present study focused on internal consistency and internal structural validity evidence. Based on the factor loadings obtained from the three-factor ESEM analysis (see Table S3), McDonald’s *ω* coefficients and 95% confidence intervals were calculated for each DASS-21 subscale across the ten countries/regions. The results demonstrated good internal consistency, with *ω* values exceeding 0.70 for each individual country/region (see Table S4).

**Table 1:** Psychometric properties (Mean, Standard Deviation, Skewness, Kurtosis) of depression items across ten countries/regions.

Region	**Item 3**			**Item 5**			**Item 10**			**Item 13**			**Item 16**			**Item 17**			**Item 21**		
	M (SD)	S	K	M (SD)	S	K	M (SD)	S	K	M (SD)	S	K	M (SD)	S	K	M (SD)	S	K	M (SD)	S	K
Mainland China	0.57 (0.71)	1.18	1.23	0.75 (0.76)	0.89	0.58	0.58 (0.74)	1.29	1.46	0.62 (0.70)	1.06	1.16	0.47 (0.67)	1.46	2.05	0.37 (0.65)	1.92	3.85	0.33 (0.62)	2.09	4.72
Ghana	0.77 (0.77)	0.82	0.30	0.75 (0.77)	0.86	0.38	0.71 (0.87)	1.17	0.68	0.77 (0.83)	0.94	0.31	0.71 (0.80)	0.99	0.45	0.65 (0.83)	1.26	1.01	0.66 (0.85)	1.26	0.94
Hong Kong	0.65 (0.82)	1.09	0.36	1.17 (0.99)	0.36	−0.96	0.77 (0.94)	0.96	−0.16	0.76 (0.86)	0.90	−0.03	0.62 (0.83)	1.19	0.55	0.36 (0.75)	2.15	3.86	1.44 (0.78)	1.84	2.78
Indonesia	0.95 (0.72)	0.60	0.55	1.07 (0.67)	0.52	0.77	0.72 (0.78)	0.93	0.40	0.96 (0.72)	0.74	0.98	0.77 (0.71)	0.70	0.42	0.64 (0.80)	1.14	0.72	0.39 (0.67)	1.85	3.32
India	0.74 (0.81)	0.85	0.03	0.98 (0.90)	0.56	−0.54	0.81 (0.88)	0.81	−0.28	0.86 (0.85)	0.68	−0.35	0.80 (0.88)	0.91	0.05	0.87 (0.97)	0.83	−0.39	0.78 (0.98)	0.98	−0.23
Malaysia	0.73 (0.86)	0.99	0.18	1.12 (0.98)	0.46	−0.82	0.66 (0.92)	1.21	0.37	0.78 (0.91)	0.94	−0.10	0.76 (0.90)	0.95	−0.08	0.67 (0.91)	1.19	0.41	0.47 (0.81)	1.69	1.98
Spain	1.32 (1.10)	0.21	−1.29	1.40 (1.13)	0.11	−1.38	1.41 (1.21)	0.12	−1.54	1.66 (1.13)	−0.17	−1.37	1.19 (1.15)	0.39	−1.31	1.17 (1.19)	0.45	−1.36	0.97 (1.18)	0.75	−1.05
Taiwan	0.78 (0.89)	0.91	−0.10	0.89 (0.89)	0.66	−0.49	0.91 (0.95)	0.72	−0.53	0.90 (0.95)	0.76	−0.45	0.76 (0.90)	0.99	0.05	0.64 (0.89)	1.19	0.37	0.71 (0.91)	1.05	0.05
Thailand	0.54 (0.70)	1.24	1.41	0.79 (0.68)	0.57	0.32	0.72 (0.83)	1.07	0.63	0.73 (0.76)	0.89	0.50	0.76 (0.77)	0.88	0.53	0.48 (0.72)	1.58	2.28	0.36 (0.67)	2.08	4.40
Vietnam	0.59 (0.70)	1.02	0.74	0.99 (0.76)	0.46	−0.07	0.58 (0.77)	1.25	1.02	0.75 (0.77)	0.76	−0.02	0.66 (0.73)	0.93	0.49	0.52 (0.73)	1.30	1.16	0.36 (0.70)	1.93	3.44
The whole students	0.69 (0.81)	1.07	0.58	0.89 (0.86)	0.76	−0.06	0.72 (0.88)	1.12	0.47	0.79 (0.86)	0.97	0.33	0.63 (0.82)	1.23	0.91	0.53 (0.82)	1.57	1.74	0.53 (0.82)	1.63	2.14

**Notes.**

M Mean SD Standard Deviation S Skewness K Kurtosis

**Table 2:** Psychometric properties (Mean, Standard Deviation, Skewness, Kurtosis) of anxiety items across ten countries/regions.

Region	**Item 2**			**Item 4**			**Item 7**			**Item 9**			**Item 15**			**Item 19**			**Item 20**		
	M (SD)	S	K	M (SD)	S	K	M (SD)	S	K	M (SD)	S	K	M (SD)	S	K	M (SD)	S	K	M (SD)	S	K
Mainland China	0.73 (0.72)	0.84	0.66	0.42 (0.64)	1.58	2.57	0.44 (0.65)	1.52	2.37	1.12 (0.88)	0.49	−0.40	0.44 (0.66)	1.56	2.54	0.44 (0.66)	1.52	2.39	0.43 (0.64)	1.52	2.39
Ghana	0.63 (0.76)	1.08	0.76	0.58 (0.78)	1.30	1.16	0.69 (0.82)	1.02	0.37	1.03 (0.95)	0.65	−0.47	0.74 (0.81)	0.97	0.43	0.73 (0.83)	0.99	0.38	0.83 (0.91)	0.94	0.04
Hong Kong	1.04 (0.91)	0.45	−0.73	0.40 (0.67)	1.62	2.01	0.33 (0.64)	2.02	3.59	1.07 (0.98)	0.46	−0.89	0.51 (0.80)	1.52	1.57	0.42 (0.71)	1.73	2.51	0.49 (0.78)	1.46	1.27
Indonesia	1.07 (0.80)	0.39	−0.29	0.50 (0.67)	1.22	1.18	0.64 (0.74)	1.02	0.64	1.35 (0.89)	0.30	−0.60	1.11 (0.82)	0.59	0.07	0.74 (0.75)	0.78	0.20	0.84 (0.84)	0.74	−0.17
India	1.04 (0.92)	0.52	−0.62	0.41 (0.66)	1.54	1.73	0.49 (0.74)	1.42	1.41	1.04 (0.95)	0.55	−0.67	0.70 (0.81)	0.96	0.21	0.68 (0.84)	1.00	0.10	0.76 (0.88)	0.94	−0.01
Malaysia	1.07 (1.02)	0.51	−0.93	0.42 (0.74)	1.78	2.50	0.58 (0.87)	1.37	0.87	0.97 (1.05)	0.67	−0.84	0.62 (0.86)	1.33	0.96	0.68 (0.91)	1.15	0.30	0.60 (0.91)	1.43	0.98
Spain	0.74 (0.98)	1.03	−0.21	0.75 (1.02)	1.08	−0.17	0.86 (1.06)	0.89	−0.60	1.41 (1.18)	0.12	−1.49	1.01 (1.13)	0.65	−1.04	1.15 (1.16)	0.44	−1.32	1.23 (1.15)	0.33	−1.36
Taiwan	0.98 (0.90)	0.63	−0.40	0.69 (0.89)	1.11	0.28	0.65 (0.87)	1.15	0.31	1.07 (0.96)	0.50	−0.76	0.74 (0.91)	0.99	−0.02	0.73 (0.90)	1.02	0.03	0.77 (0.94)	0.92	−0.31
Thailand	0.59 (0.70)	1.06	0.93	0.33 (0.62)	2.15	4.49	0.47 (0.68)	1.50	2.26	1.05 (0.84)	0.49	−0.31	0.48 (0.65)	1.34	1.89	0.48 (0.70)	1.54	2.45	0.48 (0.70)	1.45	1.89
Vietnam	0.74 (0.78)	0.75	−0.18	0.36 (0.58)	1.39	0.92	0.51 (0.72)	1.25	0.81	.0.70 (0.80)	0.91	0.09	0.38 (0.61)	1.61	2.47	0.51 (0.69)	1.19	0.79	0.53 (0.72)	1.19	0.73
The whole students	0.81 (0.82)	0.83	0.15	0.47 (0.72)	1.58	2.15	0.51 (0.75)	1.47	1.70	1.11 (0.94)	0.50	−0.65	0.57 (0.79)	1.38	1.37	0.57 (0.79)	1.37	1.30	0.59 (0.82)	1.35	1.14

**Notes.**

M Mean SD Standard Deviation S Skewness K Kurtosis

**Table 3:** Psychometric properties (Mean, Standard Deviation, Skewness, Kurtosis) of stress items across ten countries/regions.

Region	**Item 1**			**Item 6**			**Item 8**			**Item 11**			**Item 12**			**Item 14**			**Item 18**		
	M (SD)	S	K	M (SD)	S	K	M (SD)	S	K	M (SD)	S	K	M (SD)	S	K	M (SD)	S	K	M (SD)	S	K
Mainland China	0.84 (0.73)	0.76	0.75	0.52 (0.68)	1.29	1.73	0.80 (0.77)	0.80	0.38	0.67 (0.72)	0.98	0.91	0.64 (0.72)	1.04	0.97	0.77 (0.76)	0.87	0.60	0.59 (0.71)	1.17	1.31
Ghana	0.63 (0.64)	0.75	0.60	0.88 (0.88)	0.75	−0.22	0.82 (0.85)	0.87	0.11	0.73 (0.80)	0.97	0.53	0.85 (0.85)	0.87	0.25	0.73 (0.82)	0.96	0.27	0.60 (0.77)	1.33	1.58
Hong Kong	0.82 (0.85)	0.77	−0.20	0.65 (0.83)	1.11	0.41	1.21 (0.99)	0.29	−0.98	0.86 (0.91)	0.74	−0.42	0.97 (0.95)	0.63	−0.61	0.87 (0.91)	0.73	−0.41	0.72 (0.89)	1.04	0.18
Indonesia	1.09 (0.79)	0.54	0.08	0.98 (0.71)	0.51	0.42	1.05 (0.77)	0.50	0.09	1.04 (0.81)	0.42	−0.32	0.93 (0.78)	0.57	−0.01	0.80 (0.73)	0.73	0.55	1.26 (0.80)	0.40	-0.17
India	0.73 (0.74)	0.86	0.58	0.91 (0.86)	0.64	−0.34	0.80 (0.86)	0.73	−0.41	0.84 (0.83)	0.69	−0.42	0.90 (0.86)	0.68	−0.27	0.75 (0.83)	0.84	−0.11	0.60 (0.77)	1.14	0.61
Malaysia	0.86 (0.93)	0.83	−0.26	0.67 (0.87)	1.16	0.48	1.01 (0.99)	0.57	−0.80	0.76 (0.97)	1.05	−0.05	0.77 (0.93)	0.98	−0.10	0.76 (0.90)	0.98	0.02	1.25 (1.06)	0.31	-1.13
Spain	1.69 (1.08)	−0.20	−1.26	1.43 (1.10)	0.07	−1.32	1.60 (1.15)	−0.14	−1.41	1.83 (1.09)	−0.45	−1.11	1.88 (1.07)	−0.47	−1.08	1.00 (1.00)	0.68	−0.65	1.55 (1.10)	−0.06	−1.32
Taiwan	0.92 (0.89)	0.61	−0.53	0.78 (0.91)	0.86	−0.32	1.14 (0.96)	0.36	−0.90	0.97 (0.94)	0.65	−1.52	1.07 (0.94)	0.46	−0.76	1.02 (0.91)	0.52	−0.63	0.94 (0.93)	0.74	−0.34
Thailand	0.72 (0.68)	0.79	0.88	0.58 (0.68)	1.00	0.68	0.96 (0.82)	0.55	−0.25	0.66 (0.71)	0.89	0.55	0.85 (0.90)	0.90	0.07	0.84 (0.76)	0.74	0.38	0.64 (0.74)	1.01	0.62
Vietnam	0.86 (0.74)	0.53	−0.09	0.64 (0.74)	0.92	0.28	0.85 (0.85)	0.71	−0.30	0.67 (0.70)	0.77	0.11	0.73 (0.78)	0.85	0.15	0.82 (0.79)	0.61	−0.35	1.07 (0.93)	0.55	−0.53
The whole students	0.91 (0.83)	0.78	0.18	0.69 (0.83)	1.09	0.59	0.95 (0.89)	0.69	−0.29	0.83 (0.87)	0.89	0.11	0.85 (0.89)	0.88	0.01	0.82 (0.82)	0.83	0.18	0.79 (0.88)	0.97	0.20

**Notes.**

M Mean SD Standard Deviation S Skewness K Kurtosis

**Table 4:** Goodness-of-fit indices for alternative factor structures of the DASS-21 across ten countries/regions.

Region	Unidimensional model					Oblique three-factor model					Three-factor ESEM model					Bifactor ESEM model				
	*χ*^2^(df)	CFI	TLI	RMSEA	SRMR	*χ*^2^(df)	CFI	TLI	RMSEA	SRMR	*χ*^2^(df)	CFI	TLI	RMSEA	SRMR	*χ*^2^(df)	CFI	TLI	RMSEA	SRMR
Mainland China	4,896.00 (189)	0.877	0.863	0.113	0.042	4,490.94 (186)	0.888	0.873	0.109	0.040	1,250.64 (150)	0.996	0.994	0.064	0.020	721.75 (132)	0.989	0.982	0.051	0.015
Ghana	327.29 (189)	0.908	0.898	0.090	0.039	309.05 (186)	0.913	0.902	0.088	0.038	151.64 (150)	0.995	0.994	0.061	0.027	104.03 (132)	0.997	0.995	0.053	0.023
Hong Kong	798.01 (189)	0.837	0.818	0.124	0.053	565.28 (186)	0.875	0.859	0.109	0.044	221.05 (150)	0.994	0.992	0.061	0.027	152.19 (132)	0.990	0.984	0.048	0.023
Indonesia	796.55 (189)	0.774	0.749	0.136	0.067	572.53 (186)	0.834	0.812	0.118	0.058	242.86 (150)	0.988	0.983	0.084	0.038	142.96 (132)	0.982	0.973	0.050	0.029
India	507.96 (189)	0.861	0.846	0.110	0.050	343.11 (186)	0.900	0.887	0.094	0.042	169.38 (150)	0.994	0.992	0.065	0.031	114.54 (132)	0.989	0.982	0.049	0.026
Malaysia	850.49 (189)	0.837	0.818	0.138	0.050	465.02 (186)	0.896	0.882	0.111	0.037	197.86 (150)	0.995	0.994	0.074	0.025	133.70 (132)	0.989	0.982	0.058	0.020
Spain	1,293.89 (189)	0.856	0.840	0.122	0.050	539.69 (186)	0.931	0.923	0.085	0.033	213.76 (150)	0.997	0.996	0.057	0.021	140.2 (132)	0.993	0.989	0.045	0.017
Taiwan	782.87 (189)	0.908	0.898	0.113	0.029	707.84 (186)	0.917	0.906	0.108	0.028	243.16 (150)	0.998	0.997	0.076	0.016	161.2 (132)	0.993	0.989	0.059	0.014
Thailand	743.25 (189)	0.824	0.804	0.114	0.054	693.17 (186)	0.840	0.819	0.109	0.052	229.82 (150)	0.990	0.986	0.066	0.030	157.69 (132)	0.982	0.971	0.051	0.025
Vietnam	839.70 (189)	0.813	0.792	0.129	0.057	1,134.81 (186)	0.860	0.842	0.112	0.045	579.29 (150)	0.993	0.990	0.069	0.030	134.80 (132)	0.988	0.981	0.050	0.024

**Notes.**

CFI comparative fit index TLI Tucker-Lewis index RMSEA root mean square error of approximation SRMR standardized root mean square residual ESEM exploratory structural equation modeling

All countries presented significant chi-square values for all tested models (*p* < 0.001).

Several cross-loadings were also observed (see Table S3), particularly for Item 3, Item 5, and Item 13 (depression subscale), and Item 9 and Item 15 (anxiety subscale), which loaded significantly (≥0.30) on non-target factors in more than half of the regional samples. For example, Item 3 (depression subscale) exhibited cross-loadings ≥ 0.30 in six countries/regions, loading on the anxiety factor in Mainland China, Ghana and Taiwan, and on the stress factor in Indonesia, Malaysia and Vietnam. Additional details are provided in Table S5 . Notably, Item 5 and Item 9 exhibited relatively weak primary factor loadings (<0.30) in more than half of the regional samples, along with non-trivial cross-loadings on related factors. However, these patterns were confined to a small number of items rather than the subscales as a whole. Most items retained stronger loadings on their primary factors, supporting internal convergent evidence of the three-factor ESEM structure and its interpretability for within-group assessment.

In addition, inter-factor correlations were examined. The ESEM approach effectively reduced previously high correlations among the three factors, from values above 0.90 to a more moderate range of 0.44−0.66 (see Table S6), and no near-unity correlations were observed. These results provide descriptive structural evidence of factor differentiation that was not achieved with the CFA model.

### Measurement invariance

The configural invariance model, estimated using each randomly selected 15% Mainland China subsample (*n* = 941) combined with the other nine samples, yielded acceptable fit indices across five iterations (CFI = 0.996, RMSEA = 0.066−0.067, and SRMR = 0.025−0.026; see Table 5), indicating that the factor structure of the DASS-21 was consistent across cultural contexts. Metric and scalar invariance were subsequently tested. With respect to metric invariance (*i.e.,* equal factor loadings), acceptable changes in model fit were consistently observed across the five subsampling iterations (ΔCFI = −0.003 >−0.01; ΔRMSEA = 0.010−0.012 <0.015; ΔSRMR = 0.019−0.020 <0.03; see Table 5), supporting equivalence of factor loadings across the ten samples.

In contrast, scalar invariance (*i.e.,* equal item intercepts) was not supported. Imposing item intercept constraints resulted in a substantial decline in model fit, with ΔCFI values ranging from −0.097 to −0.090 (exceeding the −0.01 cutoff) and ΔRMSEA values ranging from 0.015 to 0.019 (exceeding 0.015). These fit changes exceeded Chen’s recommended thresholds for acceptable invariance (Chen, 2007), suggesting that item intercepts were not equivalent across the ten samples.

**Table 5:** Fit indices for measurement invariance testing of the DASS-21 across 10 regional groups using three-factor ESEM.

Model	*χ* ^2^	df	Model comparison	Δ*χ*^2^	Δdf	CFI	ΔCFI	RMSEA	ΔRMSEA	SRMR	ΔSRMR
***Iteration 1: Random 15% subsample of the Mainland China sample combined with the nine other regions***											
M0	2,079.00	1,500	–	–	–	0.996	–	0.066	–	0.026	–
M1	6,446.37	1,986	M1–M0	4,367.37	486	0.993	−0.003	0.077	0.011	0.045	0.019
M2	9,705.27	2,148	M2–M1	3,258.9	162	0.896	−0.097	0.094	0.017	0.045	0
***Iteration 2: Random 15% subsample of the Mainland China sample combined with the nine other regions***											
M0	2,157.71	1,500	–	–	–	0.996	–	0.066	–	0.025	–
M1	6,812.53	1,986	M1–M0	4,654.82	486	0.993	−0.003	0.078	0.012	0.045	0.020
M2	10,276.46	2,148	M2–M1	3,463.93	162	0.899	−0.094	0.093	0.015	0.045	0
***Iteration 3: Random 15% subsample of the Mainland China sample combined with the nine other regions***											
M0	2,160.78	1,500	–	–	–	0.996	–	0.067	–	0.026	–
M1	6,664.71	1986	M1–M0	4,503.93	486	0.993	−0.003	0.077	0.010	0.045	0.019
M2	9,923.01	2,148	M2–M1	3,258.30	162	0.900	−0.093	0.094	0.017	0.054	0.009
***Iteration 4: Random 15% subsample of the Mainland China sample combined with the nine other regions***											
M0	2,237.39	1,500	–	–	–	0.996	–	0.067	–	0.026	–
M1	6,775.05	1,986	M1–M0	4,537.66	486	0.993	−0.003	0.077	0.010	0.045	0.019
M2	9,958.41	2,148	M2–M1	3,183.36	162	0.903	−0.090	0.095	0.018	0.045	0
***Iteration 5: Random 15% subsample of the Mainland China sample combined with the nine other regions***											
M0	2,232.24	1,500	–	–	–	0.996	–	0.066	–	0.026	–
M1	6,834.48	1,986	M1–M0	4,602.24	486	0.993	−0.003	0.077	0.011	0.045	0.019
M2	9,813.28	2,148	M2–M1	2,978.80	162	0.899	−0.094	0.096	0.019	0.045	0

**Notes.**

CFI comparative fit index RMSEA root mean square error of approximation SRMR standardized root mean square residual M0 baseline model M1 a factor-loading constrained model M2 a factor-loading and item-intercept constrained

To clarify the potential sources of the observed cross-national non-invariance, supplementary invariance analyses were conducted across language version and several demographic and contextual variables. First, language-based invariance was examined across the seven language versions. The results supported configural invariance (CFI = 0.991, RMSEA = 0.047, and SRMR = 0.025; see Table S7) and metric invariance (ΔCFI = −0.007 >−0.01; ΔRMSEA = 0.006 <0.015; ΔSRMR = 0.019 <0.03). However, scalar invariance was again not supported, with changes in fit indices exceeding recommended cutoffs (ΔCFI = −0.018 <−0.01; ΔRMSEA = 0.022 >0.015; ΔSRMR = 0.015), indicating non-equivalence at the intercept level across language versions.

However, invariance analyses across demographic and contextual variables yielded a different pattern. More specifically, invariance was tested across age (≤20 *vs.* ≥21 years), gender (men *vs.* women), data collection mode (online *vs.* offline), and pandemic timing (onset *vs.* post-COVID-19). Scalar invariance was supported across all these subgroups, with ΔCFI values ranging from −0.004 to 0, ΔRMSEA values ranging from 0 to 0.005, and ΔSRMR values ranging from 0 to 0.003 (see Table 6), indicating that item intercepts were equivalent. These findings suggest that the DASS-21 performs consistently across different age and gender groups, as well as under varying data collection conditions and pandemic contexts.

**Table 6:** Fit indices for age, gender, data collection mode and pandemic timing measurement invariance in three-factor ESEM.

**Model fit indices for online and offline groups**											
	*χ* ^2^	df	CFI	TLI	RMSEA	SRMR					
Data set (N)											
Online (10,986)	3,455.7	150	0.990	0.986	0.045	0.020					
Offline (1,073)	451.51	150	0.990	0.985	0.043	0.026					
**Multi-group comparison across online and offline groups**											
Model	*χ* ^2^	df	Model comparison	Δ*χ*^2^	Δdf	CFI	ΔCFI	RMSEA	ΔRMSEA	SRMR	ΔSRMR
M0	3,264.34	300				0.992		0.040		0.020	
M1	2,872.06	354	M1–M0	−392.28	54	0.993	0.001	0.034	−0.007	0.024	0.004
M2	3,147.3	32	M2–M1	275.24	−3.2	0.992	−0.001	0.035	0.001	0.024	0
**Model fit indices for onset and post-COVID-19 groups**											
	*χ* ^2^	df	CFI	TLI	RMSEA	SRMR					
Data set (N)											
Onset (3,401)	3,704.44	150	0.990	0.986	0.044	0.020					
Post-COVID-19 (8,658)	2,726.22	150	0.987	0.982	0.045	0.022					
**Multi-group comparison across onset and post-COVID-19 groups**											
Model	*χ* ^2^	df	Model comparison	Δ*χ*^2^	Δdf	CFI	ΔCFI	RMSEA	ΔRMSEA	SRMR	ΔSRMR
M0	3,986.82	300				0.990		0.045		0.021	
M1	6,852.05	354	M1–M0	2,865.23	54	0.982	−0.008	0.055	0.01	0.031	0.01
M2	8,536.98	372	M2–M1	1,684.93	18	0.978	−0.004	0.060	0.005	0.033	0.002
**Model fit indices for two age groups (<20 and ≥21 years)**											
	*χ* ^2^	df	CFI	TLI	RMSEA	SRMR					
Data set (*n*)											
<=20 (7,199)	2,175.78	150	0.989	0.985	0.043	0.020					
>=21 (4,860)	1,683.64	150	0.990	0.986	0.046	0.020					
**Multi-group comparison across age groups**											
Model	*χ* ^2^	df	Model comparison	Δ*χ*^2^	Δdf	CFI	ΔCFI	RMSEA	ΔRMSEA	SRMR	ΔSRMR
M0	3,886.26	300				0.990		0.045		0.020	
M1	2,608.48	354	M1–M0	−1277.78	54	0.994	0.004	0.033	−0.012	0.022	0.002
M2	2,839.68	372	M2–M1	231.2	18	0.993	−0.001	0.033	0	0.023	0.003
**Model fit indices for men and women**											
	*χ* ^2^	df	CFI	TLI	RMSEA	SRMR					
Data set (N)											
Men (4,414)	1,539.51	150	0.990	0.986	0.046	0.021					
Women (7,645)	2,320.01	150	0.990	0.986	0.044	0.020					
**Multi-group comparison across men and women**											
Model	*χ* ^2^	df	Model comparison	Δ*χ*^2^	Δdf	CFI	ΔCFI	RMSEA	ΔRMSEA	SRMR	ΔSRMR
M0	3,847.99	300				0.990		0.044		0.020	
M1	2,829.39	354	M1–M0	−1018.6	54	0.993	0.003	0.034	−0.01	0.023	0.003
M2	2,922.77	372	M2–M1	93.38	18	0.993	0	0.034	0	0.024	0.001

**Notes.**

CFI comparative fit index TLI Tucker-Lewis index RMSEA root mean square error of approximation SRMR standardized root mean square residual M0 baseline model M1 a factor-loading constrained model M2 a factor-loading and item-intercept constrained

## Discussion

Although the DASS-21 has been widely adopted, its factor structure has remained inconsistent across studies, and prior validation research has rarely employed modeling approaches capable of accommodating cross-loadings or systematically testing measurement equivalence across culturally diverse samples. To address these limitations, the present study was designed to identify the factor structure and examine the measurement invariance of the DASS-21 among university students across ten diverse countries/regions. Through systematic model comparisons, the findings supported a three-factor ESEM model as the optimal fit, demonstrating satisfactory internal consistency and providing acceptable structural validity evidence. Measurement invariance analyses indicated configural and metric invariance across all ten countries/regions and language versions, whereas scalar invariance was not supported, suggesting potential cultural, linguistic, or other influences on item interpretation. Importantly, scalar invariance was observed across age, gender, data collection mode, and pandemic timing, indicating that the lack of scalar invariance was unlikely to reflect demographic artifacts.

### Structural validity findings: from traditional CFA to ESEM

Despite being the traditional structure of the DASS-21 (Lovibond & Lovibond, 1995a), the oblique three-factor CFA model demonstrated inadequate fit in a majority of the countries/regions examined (*i.e.,* Mainland China, Hong Kong, Indonesia, India, Malaysia, Taiwan, Thailand and Vietnam). A critical limitation was the presence of exceptionally high inter-factor correlations, frequently exceeding 0.90 in countries/regions such as Mainland China, Ghana, and Taiwan. While some overlap is expected given the shared component of negative affect in the tripartite model (Clark & Watson, 1991), correlations of this magnitude raise serious concerns about the distinctiveness of depression, anxiety, and stress as separate constructs across cultural contexts.

The three-factor ESEM model addressed these limitations by allowing cross-loadings, which provided a more realistic representation of item-factor relationships. This flexibility reduced previously inflated inter-factor correlations to a more interpretable range (0.44−0.66), supporting clear factor differentiation. The ESEM analysis helped explain why the traditional CFA model struggled to fit the data adequately: several items demonstrated substantial cross-loadings (≥ 0.30) on non-target factors in more than half of the countries/regions. More specifically, depression Item 3 (*“I couldn’t seem to experience any positive feeling at all”*), Item 5 (*“I found it difficult to work up the initiative to do things”*), and Item 13 (*“I felt down-hearted and blue”*), along with anxiety Item 9 (*“I was worried about situations in which I might panic and make a fool of myself”*) and Item 15 (*“I felt I was close to panic”*), loaded onto multiple factors, pointing to notable factor overlap. Moreover, Item 5 and Item 9 further exhibited relatively weak primary factor loadings (<0.30) alongside non-trivial cross-loadings on related factors, suggesting not only overlapping latent dimensions but also limited construct specificity at the item level.

These patterns suggest that specific items capture overlapping aspects of psychological distress, a possibility that is obscured under the restrictive zero cross-loading assumption of CFA. Furthermore, the item-level findings for Item 9 and Item 15 align with prior research identifying Item 9, Item 15, and Item 20 as forming a distinct “panic-related” factor (Alvarenga et al., 2024). This convergence suggests that these items may tap a narrower physiological-arousal construct that is conceptually distinct from the broader anxiety dimension represented in the original DASS-21 structure. Consequently, constraining these items to load exclusively on a single anxiety factor, as required under CFA, may force together items that capture conceptually separate aspects of anxiety experience, thereby contributing to the model misspecification commonly observed in CFA-based evaluations of the DASS-21.

Taken together, these findings underscore the value of ESEM for modeling the complex and intertwined nature of depression, anxiety, and stress symptoms, particularly in cross-cultural applications of the DASS-21. Consistent with previous studies demonstrating the superior performance of ESEM relative to CFA across diverse populations (Iannattone et al., 2024; Zhou et al., 2024), the present study’s findings further support Marsh & Alamer’s (2024) argument for adopting flexible modeling approaches in psychological assessment. By more accurately capturing symptom overlap and multidimensionality across cultural contexts, the three-factor ESEM model provided a robust foundation for subsequent measurement invariance testing.

### Cross-cultural measurement invariance

Measurement invariance testing in the present study supported configural and metric invariance, but not scalar invariance, across the ten samples. This finding differs from some prior cross-cultural investigations of the DASS-21 which reported scalar invariance across narrower cultural groupings or fewer countries (Castillo-Díaz & Periañez, 2025; Mellor et al., 2014; Scholten et al., 2017). The present findings indicated that while the overall factor structure and the relationships between items and latent factors of the DASS-21 were stable across cultures, more stringent forms of equivalence, particularly scalar invariance, are not consistently achieved when a broad and diverse set of cultural and linguistic contexts is considered

Scalar invariance was not supported, indicating that item intercepts differed across countries/regions. Consequently, direct comparisons of latent means across countries/regions are not warranted. The lack of scalar invariance indicates that students from different backgrounds may interpret and endorse symptom severity differently. These differences may reflect cultural and linguistic differences, or response styles.

Cultural differences across the ten sampled countries/regions likely explain the observed scalar non-invariance, primarily by shaping the thresholds at which individuals endorse symptoms rather than by altering the underlying factor structure. Notably, Spain exhibits a more individualistic tendency than the other nine countries/regions, a cultural orientation characterized by the psychologization of distress (de laTorre Luque et al., 2020; Ryder et al., 2008). In such a framework, distress is commonly conveyed through emotional, cognitive, or behavioral symptoms, such as sadness, worry, or irritability (Ryder, Yang & Heine, 2002). Consequently, the psychologization of distress in Spain may result in lower thresholds for endorsing psychological symptoms. Consistent with this interpretation, the Spanish sample demonstrated higher symptom scores and more negatively skewed distributions, which likely reflect a greater tendency to endorse distress-related items. This pattern aligns with the broader tendency in Western cultures to conceptualize and communicate distress through explicit emotional and cognitive symptoms (Ryder et al., 2008).

Linguistic factors further help explain the absence of scalar invariance. Language-based invariance analyses also supported metric but not scalar invariance, indicating that semantic differences may influence response thresholds. For example, Item 13 (*“down-hearted and blue”*) contains an idiomatic expression whose metaphorical meaning may not be consistently accessible across non-English-speaking contexts, potentially reflecting differing emotional intensities rather than depressive affect. Similarly, Item 9 (*“make a fool of myself”*) denotes embarrassment in English but may be more strongly associated with loss of face in East Asian cultures. Such semantic non-equivalence is consistent with sociolinguistic perspectives suggesting that emotion-related vocabulary is learned and refined within specific linguistic and developmental contexts (Lindquist, MacCormack & Shablack, 2015), and offers a coherent explanation for the observed lack of scalar invariance.

Importantly, scalar invariance was established across key demographic and contextual variables, including age, gender, data collection mode, and pandemic timing, which aligns with prior research (Iannattone et al., 2024; Lu et al., 2018; Zhang et al., 2024; ). These findings suggest that the DASS-21 functions equivalently across these demographic and contextual groupings, reinforcing the interpretation that the lack of scalar invariance across regional clusters is more likely attributable to cultural and linguistic influences rather than to demographic or procedural variation. This distinction underscores the importance of accounting for cultural and linguistic context when interpreting DASS-21 scores among international samples.

### Implications and future directions

Building on these findings, several directions for future research warrant further elaboration. First, given that the ESEM model provided a better fit than traditional CFA by accommodating cross-loadings, future studies should further examine the role of cross-loading items in shaping the latent structure of psychological distress, and evaluate whether alternative modeling approaches (*e.g.*, bifactor-ESEM) may better capture this complexity. Second, the lack of scalar invariance across countries/regions highlights the need to more clearly identify the sources of measurement non-equivalence, particularly cultural and linguistic influences on item interpretation. Future research could pursue item-level analyses or apply alternative approaches such as alignment optimization or Bayesian approximate invariance to pinpoint and accommodate non-equivalent items, thereby enabling more defensible cross-national comparisons. Third, the present study established cross-national measurement non-invariance at a single timepoint, but whether this pattern is itself stable needs further verification. Future research employing longitudinal or test–retest designs could examine whether the DASS-21’s factor structure and its cross-national non-equivalence persist, attenuate, or vary over time, thereby clarifying whether the observed cultural and linguistic influences reflect enduring or context-dependent characteristics.

These findings also carry meaningful implications for practitioners working with university students, including counselors, psychologists, mental health professionals, and student support staff who rely on standardized self-report instruments in assessment and intervention planning. The evidence supporting the structural validity of the DASS-21 across diverse university populations indicates that the scale can serve as a useful tool for within-group screening and monitoring of depression, anxiety, and stress symptoms. However, the absence of scalar invariance means that observed score differences across countries/regions may reflect measurement artifacts rather than true differences in symptom levels. Practitioners working in multicultural or international settings should therefore avoid treating DASS-21 scores as directly comparable across cultural or linguistic contexts. Instead, the instrument is best used as one component of a broader, culturally informed evaluation that integrates contextual knowledge, clinical judgment, and complementary assessment methods.

### Strengths and limitations

The present study offers several strengths. First, it is based on a large international sample of 12,059 university students drawn from ten culturally diverse countries/regions, with each country/region contributing more than 400 participants. This design provided strong statistical power and enabled systematic cross-national evaluation of the DASS-21 across markedly different cultural contexts, which has rarely been undertaken at this scale in prior validation work. Second, a rigorous model comparison was conducted, encompassing both traditional structures (unidimensional, oblique three-factor, bifactor CFA) and advanced structures (three-factor ESEM, bifactor ESEM). This comprehensive analytic approach allowed for the evaluation of cross-loading patterns that are obscured under the conventional CFA framework, providing a more accurate representation of the interrelations among the depression, anxiety, and stress dimensions. Third, the present study represents one of the first to systematically test measurement invariance of the DASS-21 using an ESEM framework across a large number of culturally diverse samples, addressing a critical methodological gap in prior cross-cultural validation research. Fourth, supplementary invariance analyses across demographic and contextual variables (age, gender, data collection mode, and pandemic timing) confirmed that the observed cross-national non-invariance was unlikely to reflect demographic or procedural artifacts, strengthening the substantive interpretation of cultural and linguistic influences.

However, several limitations should be noted. The use of convenience and snowball sampling may limit the representativeness of the sample within each country/region because it may not fully capture the demographic or psychological diversity of the broader student population. Notably, participants from Mainland China accounted for 52.06% of the total sample, indicating a substantial imbalance in regional representation. Although measurement invariance analyses were conducted using repeated random subsampling (15% of the Chinese sample across five iterations) to mitigate this issue, this approach may not fully eliminate potential bias. The gender distribution was also imbalanced, with women comprising 63.4% of the sample. In addition, gender was assessed using a binary classification (males/females), which excluded gender-diverse individuals and does not fully reflect current standards in psychological research.

Additionally, the present study relied solely on previously validated versions of the DASS-21, without conducting any adaptation steps or harmonization checks across language versions (*e.g.*, translation back-translation procedures or expert panel review to verify semantic equivalence of items across languages), which may potentially introduce measurement bias. Reliance on self-report data across culturally diverse countries/regions also introduces the risk of response bias, as cultural norms and language may influence item interpretation. Moreover, the study relied on cross-sectional secondary analysis, leaving the temporal aspects of the DASS-21’s psychometric properties beyond the scope of the present study’s design. Finally, although the convergence of demographic and contextual invariance with cross-national non-invariance points to cultural and linguistic factors as the primary source of measurement non-equivalence, this attribution remains inferential. The present design cannot directly isolate the specific cultural or linguistic mechanisms involved, and the interpretation should therefore be regarded as preliminary pending confirmation in future cross-national studies.

## Conclusions

Despite the widespread use of the DASS-21 in research, evidence regarding its cross-cultural factor structure and measurement invariance using modeling approaches that accommodate cross-loadings remains limited. To address this gap, the present study employed ESEM in a large multi-country/region sample of university students. The results supported a three-factor ESEM structure across all countries/regions, with consistent evidence of internal reliability as well as configural and metric invariance. The finding that scalar invariance was achieved across demographic and methodological factors, but not across cultural and linguistic groups, highlights the potential influence of cultural and language-related factors on responses to the DASS-21. However, these findings should be interpreted in light of several limitations, including sampling imbalance, the cross-sectional design, and the reliance on self-report measures. Taken together, these findings underscore the value of flexible modeling approaches for assessing psychological distress in cross-cultural research and provide robust psychometric evidence for the DASS-21 within an ESEM framework among university students. At the same time, they point to important constraints in making direct cross-cultural comparisons of psychological distress.

##  Supplemental Information

10.7717/peerj.21745/supp-1 Supplemental Information 1 The data of DASS-21 among ten regions

10.7717/peerj.21745/supp-2 Supplemental Information 2 Information of participants, Factor loading, and sensitivity analysis Table S1 Information of P articipants
Table S2 Standardized Factor Loadings in the ESEM Model Across Ten Regions
Table S3 Comparison of Inter-Factor Correlations in DASS-21: Oblique Three-Factor Model vs. Three-Factor Exploratory Structural Equation Model (ESEM) Model
Table S4 Sensitivity Analysis Using Two-Stage Robust Estimation: Model Fit Across Ten Regions
Table S5 Sensitivity Analysis Using Two-Stage Robust Estimation: Measurement Invariance Across Ten Regions

10.7717/peerj.21745/supp-3 Supplemental Information 3 Checklist

## Additional Information and Declarations

### Data Availability

The following information was supplied regarding data availability: The raw data is available in the Supplemental File.

## References

[ref-1] Adu P, Popoola T, Iqbal N, Medvedev ON, Simpson CR (2025). Validating the Depression Anxiety Stress Scales (DASS-21) across Germany, Ghana, India, and New Zealand using Rasch methodology. Journal of Affective Disorders.

[ref-2] Alamer A, Alrabai F (2024). The second language trait emotional intelligence (L2-TEI) scale and its relation to language engagement. Acta Psychologica.

[ref-3] Alamer A, Marsh H (2022). Exploratory structural equation modeling in second language research: an applied example using the dualistic model of passion. Studies in Second Language Acquisition.

[ref-4] Alfonsson S, Wallin E, Maathz P (2017). Factor structure and validity of the depression, anxiety and stress scale-21 in Swedish translation. Journal of Psychiatric and Mental Health Nursing.

[ref-5] Alvarenga MAS, Ali AM, Guilhermino CS, De Azevedo TG, Olímpio KF, Kersul C, Da Cunha GL, Alvares-Teodoro J, Ribeiro PCC, Mansur-Alves M, Teodoro MLM, Bandeira PFR (2024). A cross-sectional network analysis study applied to the depression anxiety stress scale-21 (DASS-21): a new factor revealed. Mental Illness.

[ref-6] Aruta J, Mateo NJ, Resurreccion RR, Galanza M, Bernardo ABI (2024). Screening psychological symptoms in Filipino university students during the COVID-19 pandemic: translation and structural validation of the Filipino version of the DASS-21. Psychology in the Schools.

[ref-7] Asparouhov T, Muthén B (2009). Exploratory structural equation modeling. Structural Equation Modeling: A Multidisciplinary Journal.

[ref-8] Bibi A, Lin MY, Zhang XC, Margraf J (2020). Psychometric properties and measurement invariance of Depression, Anxiety and Stress Scales (DASS-21) across cultures. International Journal of Psychology.

[ref-9] Bowling NA, Huang JL, Brower CK, Bragg CB (2023). The quick and the careless: the construct validity of page time as a measure of insufficient effort responding to surveys. Organizational Research Methods.

[ref-10] Browne MW, Cudeck RA (1992). Alternative ways of assessing model fit. Sociological Methods & Research.

[ref-11] Castillo-Díaz MA, Periañez CAH (2025). Dimensionality, reliability, invariance, and item analysis of the Depression, Anxiety, and Stress Scale-21 (DASS-21) in Honduran and Colombian university students. BMC Psychology.

[ref-12] Chaturvedi SK (2015). Religious, spiritual, and cultural aspects of psychiatric ethics in Hinduism. The Oxford handbook of psychiatric ethics.

[ref-13] Chen FF (2007). Sensitivity of goodness of fit indexes to lack of measurement invariance. Structural Equation Modeling: A Multidisciplinary Journal.

[ref-14] Clark LA, Watson D (1991). Tripartite model of anxiety and depression: psychometric evidence and taxonomic implications. Journal of Abnormal Psychology.

[ref-15] Cousi C, Igier V, Quintard B (2021). French cross-cultural adaptation and validation of the Quality of Life-Alzheimer’s Disease scale in Nursing Homes (QOL-AD NH). Health and Quality of Life Outcomes.

[ref-16] de laTorre Luque A, Ojagbemi A, Caballero FF, Lara E, Moreno-Agostino D, Bello T, Olaya B, Haro JM, Gureje O, Ayuso-Mateos JL (2020). Cross-cultural comparison of symptom networks in late-life major depressive disorder: Yoruba Africans and the Spanish population. International Journal of Geriatric Psychiatry.

[ref-17] Duffy A (2023). University student mental health: an important window of opportunity for prevention and early intervention. The Canadian Journal of Psychiatry.

[ref-18] Dwight AR, Briesch AM, Hoffman JA, Rutt C (2024). Systematic review of the psychometric evidence supporting use of the Depression Anxiety Stress Scales, Short Form (DASS-21) with youth. Child & Youth Care Forum.

[ref-19] Fornell C, Larcker DF (1981). Evaluating structural equation models with unobservable variables and measurement error. Journal of Marketing Research.

[ref-20] Gomez R, Stavropoulos V, Griffiths MD (2020). Confirmatory factor analysis and exploratory structural equation modelling of the factor structure of the Depression Anxiety and Stress Scales-21. PLOS ONE.

[ref-21] Healthy Minds Network (2022). Healthy minds study among colleges and universities. https://healthymindsnetwork.org/wp-content/uploads/2023/08/HMS-National-Report-2021-22_full.pdf.

[ref-22] Hinkelman JM, Luzzo DA (2007). Mental health and career development of college students. Journal of Counseling & Development.

[ref-23] Iannattone S, Mignemi G, Pivetta E, Gatta M, Sica C, Cardi V, Canale N, Spoto A, Bottesi G (2024). Are anxiety, depression, and stress distinguishable in Italian adolescents? An examination through the Depression Anxiety Stress Scales-21. PLOS ONE.

[ref-24] Ifdil I, Syahputra Y, Fadli RP, Zola N, Putri YE, Amalianita B, Rangka IB, Suranta K, Zatrahadi MF, Sugara GS, Situmorang DDB, Fitria L (2022). The depression anxiety stress scales (DASS-21); an Indonesian validation Measure of the depression anxiety stress. COUNS EDU: The International Journal of Counseling and Education.

[ref-25] Jiang L-C, Yan Y-J, Jin Z-S, Hu M-L, Wang L, Song Y, Li N-N, Su J, Wu D-X, Xiao T (2022). The depression anxiety stress scale-21 in Chinese hospital workers: reliability, latent structure, and measurement invariance across genders. Frontiers in Psychology.

[ref-26] Jovanović V, Gavrilov-Jerković V, Lazić M (2021). Can adolescents differentiate between depression, anxiety and stress? Testing competing models of the Depression Anxiety Stress Scales (DASS-21). Current Psychology.

[ref-27] Kline RB (2023). Principles and practice of structural equation modeling.

[ref-28] Li J (2023). Confucian affect (Qing) as the foundation for mutual care and moral elevation. Journal of Confucian Philosophy and Culture.

[ref-29] Lindquist KA, MacCormack JK, Shablack H (2015). The role of language in emotion: predictions from psychological constructionism. Frontiers in Psychology.

[ref-30] Liu X, Wang J (2024). Depression, anxiety, and student satisfaction with university life among college students: a cross-lagged study. Humanities and Social Sciences Communications.

[ref-31] Lovibond PF, Lovibond SH (1995a). The structure of negative emotional states: comparison of the Depression Anxiety Stress Scales (DASS) with the beck depression and anxiety inventories. Behaviour Research and Therapy.

[ref-32] Lovibond SH, Lovibond PF (1995b). Manual for the depression anxiety stress scales.

[ref-33] Lu S, Hu S, Guan Y, Xiao J, Cai D, Gao Z, Sang Z, Wei J, Zhang X, Margraf J (2018). Measurement invariance of the Depression Anxiety Stress Scales-21 across gender in a sample of Chinese university students. Frontiers in Psychology.

[ref-34] Maassen E, D’Urso ED, Van Assen MALM, Nuijten MB, De Roover K, Wicherts JM (2025). The dire disregard of measurement invariance testing in psychological science. Psychological Methods.

[ref-35] Marsh H, Alamer A (2024). When and how to use set-exploratory structural equation modelling to test structural models: a tutorial using the R package lavaan. British Journal of Mathematical and Statistical Psychology.

[ref-36] Marsh HW, Liem GAD, Martin AJ, Morin AJS, Nagengast B (2011). Methodological measurement fruitfulness of exploratory structural equation modeling (ESEM): new approaches to key substantive issues in motivation and engagement. Journal of Psychoeducational Assessment.

[ref-37] Marsh HW, Morin AJ, Parker PD, Kaur G (2014). Exploratory structural equation modeling: an integration of the best features of exploratory and confirmatory factor analysis. Annual Review of Clinical Psychology.

[ref-38] Mellor D, Vinet EV, Xu X, Hidayah Bt, Mamat N, Richardson B, Román F (2014). Factorial invariance of the DASS-21 among adolescents in four countries. European Journal of Psychological Assessment.

[ref-39] Morin AJS (2023). Exploratory structural equation modeling. Handbook of structural equation modeling.

[ref-40] Moussa MT, Lovibond PF, Laube R (2001). Psychometric properties of a Chinese version of the 21-item Depression Anxiety Stress Scales (DASS-21).

[ref-41] NCHECR Thai translation (2007). A Thai translation of DASS. https://www2.psy.unsw.edu.au/Groups/Dass/Thai/NCHECR_Thai.htm.

[ref-42] Oei TPS, Sawang S, Goh YW, Mukhtar F (2013). Using the Depression Anxiety Stress Scale 21 (DASS-21) across cultures. International Journal of Psychology.

[ref-43] Paiva U, Cortese S, Flor M, Moncada-Parra A, Lecumberri A, Eudave L, Magallón S, García-González S, Sobrino-Morras Á, Piqué IM, Mestre-Bach G, Solmi M, Arrondo G (2025). Prevalence of mental disorder symptoms among university students: an umbrella review. Neuroscience and Biobehavioral Reviews.

[ref-44] Patricia D, Diane MN, Melinda AS, Patricia A (2002). The Depression Anxiety Stress Scale-21: Spanish translation and validation with a Hispanic sample. Journal of Psychopathology and Behavioral Assessment.

[ref-45] Patrick J, Dyck M, Bramston P (2010). Depression Anxiety Stress Scale: is it valid for children and adolescents?. Journal of Clinical Psychology.

[ref-46] Pholphirul P, Srijamdee K (2025). Buddhist practices and mental health outcomes in Thailand. Journal of Religion & Health.

[ref-47] R Core Team (2025). R: a language and environment for statistical computing. https://www.r-project.org.

[ref-48] Ryder AG, Yang J, Heine SJ (2002). Somatization *vs.* psychologization of emotional distress: a paradigmatic example for cultural psychopathology. Online Readings in Psychology and Culture.

[ref-49] Ryder AG, Yang J, Zhu X, Yao S, Yi J, Heine SJ, Bagby RM (2008). The cultural shaping of depression: somatic symptoms in China, psychological symptoms in North America?. Journal of Abnormal Psychology.

[ref-50] Şahin E, Topkaya N, Gençoğlu C (2022). Severity and correlates of the symptoms of depression, anxiety, and stress in a nationally representative sample of Turkish secondary boarding school Counselors. SAGE Open.

[ref-51] Sánchez-Carracedo D, Barrada JR, López-Guimerà G, Fauquet J, Almenara CA, Trepat E (2012). Analysis of the factor structure of the Sociocultural Attitudes Towards Appearance Questionnaire (SATAQ-3) in Spanish secondary-school students through exploratory structural equation modeling. Body Image.

[ref-52] Scholten S, Velten J, Bieda A, Zhang XC, Margraf J (2017). Testing measurement invariance of the Depression, Anxiety, and Stress Scales (DASS-21) across four countries. Psychological Assessment.

[ref-53] Sinval J, Oliveira P, Novais F, Almeida CM, Telles-Correia D (2025). Exploring the impact of depression, anxiety, stress, academic engagement, and dropout intention on medical students’ academic performance: a prospective study. Journal of Affective Disorders.

[ref-54] Swami V, Maïano C, Morin AJS (2023). A guide to exploratory structural equation modeling (ESEM) and bifactor-ESEM in body image research. Body Image.

[ref-55] Tanaka S, Maulina VVR, Shanti TI, Prabowo MW (2025). Cultural diversity in beliefs regarding mental illness: comparison of Indonesian Muslims, Indonesian Christians, and Japanese non-religions. Frontiers in Psychology.

[ref-56] Tran TD, Tran T, Fisher J (2013). Validation of the Depression Anxiety Stress Scales (DASS) 21 as a screening instrument for depression and anxiety in a rural community-based cohort of northern Vietnamese women. BMC Psychiatry.

[ref-57] Udah H, Tusasiirwe S, Mugumbate R, Gatwiri K (2025). Ubuntu philosophy, values, and principles: an opportunity to do social work differently. Journal of Social Work.

[ref-58] World Health Organization (2023). Statement on the fifteenth meeting of the IHR(2005) Emergency Committee on the COVID-19 pandemic. https://www.who.int/news/item/05-05-2023-statement-on-the-fifteenth-meeting-of-the-international-health-regulations-%282005%29-emergency-committee-regarding-the-coronavirus-disease-%28covid-19%29-pandemic.

[ref-59] Yeung AY, Yuliawati L, Cheung SH (2020). A systematic review and meta-analytic factor analysis of the depression anxiety stress scales. Clinical Psychology: Science and Practice.

[ref-60] Yılmaz Koğar E, Koğar H (2022). Using a bifactor exploratory structural equation modeling framework to examine the factor structure of the Depression Anxiety and Stress Scales-21. Current Psychology.

[ref-61] Zanon C, Brenner RE, Baptista MN, Vogel DL, Rubin M, Al-Darmaki FR, Gonçalves M, Heath PJ, Liao H-Y, Mackenzie CS, Topkaya N, Wade NG, Zlati A (2021). Examining the dimensionality, reliability, and invariance of the Depression, Anxiety, and Stress Scale–21 (DASS-21) across eight countries. Assessment.

[ref-62] Zanon C, Zhao N, Topkaya N, Şahin E, Vogel DL, Ertl MM, Sanatkar S, Liao H-Y, Rubin M, Baptista MN, Mak WWS, Al-Darmaki FR, Schomerus G, Wang Y-F, Nasvytienė D (2025). Psychometric properties of the depression, anxiety, and stress scale–21 (DASS-21) across nine countries/regions. International Journal of Testing.

[ref-63] Zhang Z, Lin R, Qiu A, Wu HF, Wu S, Zhang L (2024). Application of DASS-21 in Chinese students: invariance testing and network analysis. BMC Public Health.

[ref-64] Zhou X-H, Shen Z-Z, Cao C-H, Liao X-L, Jiang X-Y, Griffiths MD, Chen I-H, Lin C-Y, Malas O (2024). Psychometric evaluation of DASS versions among Spanish and Chinese teachers using exploratory structural equation modeling (ESEM). Acta Psychologica.

[ref-65] Zuo K, Chang A (2008). A Chinese Translation of the DASS (simplified characters). https://www2.psy.unsw.edu.au/groups/dass/Chinese/Chinese%20simplified.htm.

