# Supplementary material for: A cross-sectional study examining the reliability and invariance of the Depression, Anxiety, and Stress Scale–21 (DASS-21) across ten countries/regions
Source: PeerJ. 2026 Sep 29;14:e21745. doi: 10.7717/peerj.21745 (PMC13632677; doi:10.7717/peerj.21745)
Supplement: Supplementary file 1 [file peerj-14-21745-s002.docx]

Table S1. Information of Participants.

| Region | N(%) | Age range | Original source | Time of the data collection | Ethical approval No. | Online (Platform)/ Offline | Version of DASS-21 | University |
| --- | --- | --- | --- | --- | --- | --- | --- | --- |
| Mainland China | 6278 (52.06%) | 18 - 30 | Submitted | Sep-Oct 2023 | Jiangxi Psychological Consultant  Association (IRB ref: JXSXL-2022-Jul13) | Online (*Wen Juan Xing*) | Simplified Chinese character DASS-21 | Mainland China-based universities |
| Ghana | 466 (3.87%) | 18 - 28 | https://doi.org/10.32604/ijmhp.2024.057049 | Jul-Aug 2023 | Kwame Nkrumah University of Science and Technology (CHRPE/AP/612/23) | Offline | English DASS-21 | Kwame Nkrumah University of Science and Technology |
| Hong Kong | 759 (6.29%) | 18 - 28 | https://doi.org/10.1080/1091367X.2024.2302607 | May-Aug 2023 | Survey and Behavioural Research Committee at the Chinese University of Hong Kong (Reference No. SBRE‐22‐0186) | Online (*Qualtrics*) | Traditional Chinese character DASS-21 | Hong Kong-based universities |
| Indonesia | 458 (3.81%) | 18 - 28 | https://doi.org/10.30773/pi.2022.0304 | Jul-Dec 2021 | Health Research Ethics Commission, Faculty of Nursing, Universitas Airlangga (registration number: 2318-KEPK) | Online (*Google Forms*) | Bahasa Indonesian DASS-21 | Universitas Padjadjaran in West Java and the Universitas Airlangga in East Java |
| India | 454 (3.76%) | 18 - 28 | Under revision | Mar-June 2023 | Indian Institute of Technology Bombay Review Board ( IITB-IRB/2022/045) | Online (*Google Forms*) | English DASS-21 | Universities in India |
| Malaysia | 624 (5.17%) | 18 - 28 | https://doi.org/10.1177/01632787231185845 | May-Aug 2022 | Jawatankuasa Etika Universiti Penyelidikan Melibatkan Manusia; Reference Number: JKEUPM-2022-268 | Online (*Google Forms*) | English DASS-21 | Universities in Malaysia |
| Spain | 1024 (8.49%) | 18 - 34 | not Published before | Mar-Apr 2022 | It was considered exempt by the IRB of its institution (CEIM HUAV) | Online (*Google Forms*) | Spanish DASS-21 | Universities in Catalonia region (Spain) |
| Taiwan | 973 (8.07%) | 18 - 28 | https://doi.org/10.1186/s40337-022-00701-y | Aug 2021-Apr  2022 | Institutional Review Board in the Chi Mei Medical Center (IRB Serial No.: 11007006);  Human Research Ethics Committee in the National Cheng Kung University (Approval No.: NCKU HREC-E-109-551-2) | Online (*Google Forms*) | Traditional Chinese character DASS-21 | Universities in Taiwan |
| Thailand | 413 (3.42%) | 18 - 28 | <https://doi.org/10.1177/01632787241271117> ^a^ | Jul-Aug 2023 | Human Research Ethics of National Cheng Kung University (NCKU HREC-E-110-486-2) and endorsed by the ethics committee at Mahidol University. | Online (*SurveyMonkey*) | Thai DASS-21 | Universities in Thailand |
| Vietnam | 610 (5.06%) | 19 - 27 | https://doi.org/10.1186/s41155-024-00328-9 | Apr-June 2023 | Board of Ethics in Biomedical Research in the University of Medicine and Pharmacy at Ho Chi Minh City (Number: 1080/UMP-BOARD) | Offline | Vietnamese DASS-21 | University of Medicine and Pharmacy at Ho Chi Minh City |

**Notes:** Citations for papers based on compiled data. ^a^ Although the publication does not report using the Depression, Anxiety, Stress Scale-21 (DASS-21), the authors who published this paper had collected DASS-21 in their project.

Table S2. Goodness-of-Fit Indices for Alternative Factor Structures of the DASS-21 Across Ten Regions

| Region | Unidimensional model | | | Oblique three-factor model | | | Three-factor ESEM model | | | Bifactor ESEM model | | |
| --- | --- | --- | --- | --- | --- | --- | --- | --- | --- | --- | --- | --- |
|  | χ² (df) | CFI | RMSEA | χ² (df) | CFI | RMSEA | χ² (df) | CFI | RMSEA | χ² (df) | CFI | RMSEA |
| Mainland China | 5157.55 (189) | 0.889 | 0.065 | 4757.44 (186) | 0.897 | 0.082 | 1964.84 (150) | 0.959 | 0.044 | 1379.39 (132) | 0.972 | 0.039 |
| Ghana | 378.89 (189) | 0.939 | 0.046 | 364.79 (186) | 0.943 | 0.045 | 332.48 (150) | 0.942 | 0.051 | 244.88 (132) | 0.967 | 0.043 |
| Hong Kong | 890.88 (189) | 0.868 | 0.070 | 762.57 (186) | 0.891 | 0.064 | 432.78 (150) | 0.947 | 0.050 | 359.57 (132) | 0.957 | 0.048 |
| Indonesia | 828.76 (189) | 0.832 | 0.086 | 636.87 (186) | 0.884 | 0.073 | 405.61 (150) | 0.933 | 0.061 | 361.88 (132) | 0.940 | 0.062 |
| India | 545.73 (189) | 0.896 | 0.064 | 415.19 (186) | 0.933 | 0.052 | 342.49 (150) | 0.944 | 0.053 | 244.88 (132) | 0.967 | 0.043 |
| Malaysia | 973.65 (189) | 0.863 | 0.082 | 659.06 (186) | 0.917 | 0.064 | 452.91 (150) | 0.947 | 0.057 | 375.50 (132) | 0.957 | 0.054 |
| Spain | 1598.22 (189) | 0.872 | 0.085 | 826.83 (186) | 0.942 | 0.058 | 465.12 (150) | 0.971 | 0.045 | 366.20 (132) | 0.979 | 0.042 |
| Taiwan | 1007.83 (189) | 0.920 | 0.067 | 911.36 (186) | 0.929 | 0.063 | 453.77 (150) | 0.970 | 0.046 | 370.04 (132) | 0.977 | 0.043 |
| Thailand | 588.95 (189) | 0.861 | 0.072 | 492.48 (186) | 0.896 | 0.074 | 317.03 (150) | 0.942 | 0.052 | 267.00 (132) | 0.953 | 0.050 |
| Vietnam | 857.05 (189) | 0.850 | 0.076 | 648.49 (186) | 0.896 | 0.064 | 425.32 (150) | 0.938 | 0.055 | 336.87 (132) | 0.954 | 0.050 |

**Note**: CFI, comparative fit index; RMSEA, root mean square error of approximation; ESEM, exploratory structural equation modeling. All countries presented significant chi-square values for all tested models (*p* < 0.001).

Table S3. Standardized Factor Loadings in the ESEM Model Across Ten Regions

|  | CHN | | | GHA | | | HKG | | | IDN | | | IND | | | MYS | | | ESP | | | TWN | | | THA | | | VNM | | |
| --- | --- | --- | --- | --- | --- | --- | --- | --- | --- | --- | --- | --- | --- | --- | --- | --- | --- | --- | --- | --- | --- | --- | --- | --- | --- | --- | --- | --- | --- | --- |
|  | Dep | Anx | Stress | Dep | Anx | Stress | Dep | Anx | Stress | Dep | Anx | Stress | Dep | Anx | Stress | Dep | Anx | Stress | Dep | Anx | Stress | Dep | Anx | Stress | Dep | Anx | Stress | Dep | Anx | Stress |
| Dep |  |  |  |  |  |  |  |  |  |  |  |  |  |  |  |  |  |  |  |  |  |  |  |  |  |  |  |  |  |  |
| Item 3 | .20 | .46 | .14 | .19 | .49 | .16 | .39 | .16 | .29 | .44 | .19 | .20 | .03 | -.02 | .57 | .44 | .09 | .35 | .57 | .15 | .24 | .28 | .43 | .25 | .42 | .17 | .17 | .31 | .02 | .52 |
| Item 5 | .09 | .34 | .35 | .12 | .37 | .30 | .24 | .06 | .38 | .23 | .12 | .30 | .28 | .22 | .44 | .31 | -.06 | .58 | .41 | .21 | .20 | .17 | .38 | .30 | .19 | .14 | .41 | .27 | .06 | .35 |
| Item 10 | .40 | .10 | .37 | .58 | .05 | .22 | .52 | .04 | .31 | .66 | .11 | .16 | .14 | .12 | .14 | .72 | .12 | .12 | .77 | -.01 | .19 | .40 | .04 | .50 | .50 | -.02 | .32 | .76 | .04 | .09 |
| Item 13 | .32 | .15 | .51 | .54 | .15 | .24 | .39 | .06 | .50 | .36 | .17 | .37 | .67 | -.03 | .68 | .41 | .12 | .42 | .54 | .06 | .39 | .36 | .09 | .55 | .42 | .17 | .35 | .25 | .09 | .61 |
| Item 16 | .59 | .13 | .18 | .47 | .30 | .08 | .57 | .02 | .25 | .62 | .03 | .15 | .21 | .05 | .39 | .60 | -.01 | .35 | .72 | .12 | .13 | .46 | .25 | .24 | .37 | .16 | .29 | .49 | .13 | .27 |
| Item 17 | .80 | .08 | .03 | .46 | .29 | .06 | .75 | .20 | -.13 | .82 | .05 | .03 | .40 | -.02 | .20 | .84 | .07 | .02 | .61 | .23 | .08 | .80 | .14 | .03 | .82 | .12 | -.07 | .74 | .08 | .05 |
| Item 21 | .77 | .12 | -.02 | .53 | .29 | -.02 | .71 | .08 | -.04 | .77 | .18 | -.13 | .70 | -.01 | .57 | .79 | .16 | -.05 | .72 | .14 | -.03 | .70 | .03 | .21 | .90 | .05 | -.11 | .79 | .04 | -.04 |
| Anx |  |  |  |  |  |  |  |  |  |  |  |  |  |  |  |  |  |  |  |  |  |  |  |  |  |  |  |  |  |  |
| Item 2 | -.04 | .56 | .15 | -.17 | .71 | -.01 | -.07 | .36 | .23 | .05 | .46 | .06 | -.05 | .22 | 0 | .12 | .25 | .26 | .04 | .46 | .09 | -.07 | .55 | .21 | .07 | .29 | .20 | -.02 | .33 | .24 |
| Item 4 | .16 | .71 | -.06 | .18 | .25 | .27 | .13 | .65 | -.04 | -.01 | .73 | -.15 | .06 | .43 | .26 | .07 | .70 | .02 | .13 | .61 | .08 | .30 | .60 | .02 | .06 | .74 | -.07 | .02 | .65 | .02 |
| Item 7 | .13 | .53 | .09 | -.08 | .04 | .75 | .13 | .62 | -.05 | .05 | .45 | .15 | .01 | .48 | .18 | .08 | .64 | .08 | -.05 | .70 | .11 | .37 | .57 | -.03 | .05 | .53 | .03 | -.03 | .48 | .09 |
| Item 9 | -.07 | .07 | .70 | .32 | .11 | .41 | .04 | .18 | .56 | .19 | .01 | .67 | .16 | .43 | .16 | .15 | .33 | .35 | .19 | .37 | .31 | .16 | .11 | .61 | .08 | .12 | .61 | .24 | .36 | .19 |
| Item 15 | .47 | .22 | .23 | .30 | .15 | .40 | .43 | .23 | .29 | .02 | .19 | .64 | .25 | .59 | .26 | .14 | .49 | .33 | .30 | .48 | .14 | .48 | .31 | .19 | .16 | .37 | .36 | .22 | .53 | .14 |
| Item 19 | .43 | .30 | .14 | .13 | .49 | .21 | .06 | .72 | 0 | .07 | .64 | -.07 | .07 | .40 | .06 | .16 | .67 | -.02 | .02 | .64 | .18 | .44 | .43 | .05 | .03 | .80 | -.11 | .01 | .76 | -.01 |
| Item 20 | .43 | .27 | .19 | .28 | .35 | .22 | .34 | .42 | .14 | .31 | .37 | .31 | .31 | .45 | .18 | .30 | .52 | .11 | .26 | .46 | .17 | .51 | .27 | .22 | .20 | .36 | .30 | .26 | .56 | .08 |
| Stress |  |  |  |  |  |  |  |  |  |  |  |  |  |  |  |  |  |  |  |  |  |  |  |  |  |  |  |  |  |  |
| Item 1 | -.02 | .42 | .22 | .02 | .51 | .15 | .05 | .25 | .39 | .02 | .31 | .22 | .01 | .10 | .52 | .11 | .28 | .45 | .08 | .15 | .62 | .03 | .55 | .17 | .15 | .15 | .45 | -.04 | .06 | .60 |
| Item 6 | .12 | .51 | .19 | 0 | .25 | .54 | .07 | .41 | .33 | -.04 | .16 | .54 | .10 | .47 | .21 | .07 | .17 | .57 | .14 | .28 | .38 | .31 | .38 | .25 | .06 | .17 | .46 | .20 | .19 | .35 |
| Item 8 | 0 | .28 | .53 | .07 | .19 | .56 | .02 | .19 | .54 | .19 | .29 | .51 | .05 | .60 | .22 | -.06 | .46 | .40 | .06 | .27 | .47 | -.02 | .37 | .51 | .09 | .16 | .65 | .15 | .27 | .44 |
| Item 11 | .17 | .17 | .61 | .46 | .11 | .30 | .23 | .14 | .58 | .14 | .16 | .68 | .25 | .44 | .27 | .26 | .38 | .35 | .02 | .11 | .71 | .20 | .10 | .67 | .15 | .24 | .52 | .08 | .31 | .48 |
| Item 12 | .23 | .16 | .55 | .55 | .20 | .16 | .21 | .10 | .62 | .08 | .35 | .34 | .14 | .24 | .52 | .13 | .31 | .53 | .06 | .03 | .83 | .05 | .14 | .72 | .26 | .13 | 0.4 | .08 | .14 | .68 |
| Item 14 | .11 | .11 | .48 | .48 | -.04 | .36 | .01 | .23 | .38 | .23 | .16 | .33 | .20 | .36 | .25 | .09 | .17 | .57 | .18 | .33 | .21 | .09 | .28 | .44 | .13 | .16 | .42 | .27 | .22 | .33 |
| Item 18 | .35 | .14 | .34 | .28 | .27 | .22 | .08 | .41 | .26 | .24 | .07 | .45 | .33 | .40 | .03 | .19 | .17 | .36 | .27 | .21 | .35 | .17 | .34 | .35 | .21 | .21 | .34 | .21 | .23 | .38 |

**Note:** CHN (Mainland China) GHA (Ghana) HKG (Hong Kong) IDN (Indonesia) IND (India) MYS (Malaysia) ESP (Spain) TWN (Taiwan) THA (Thailand) VNM (Vietnam)

Table S4. Internal Consistency of Depression, Anxiety and Stress Across All Regions

| Region | Deprssion | | Anxiety | | Stress | |
| --- | --- | --- | --- | --- | --- | --- |
|  | McDonald’s ω | 95% CI | McDonald’s ω | 95% CI | McDonald’s ω | 95% CI |
| Mainland China | 0.892 | [0.888, 0.896] | 0.846 | [0.840, 0.852] | 0.866 | [0.861, 0.871] |
| Ghana | 0.884 | [0.867, 0.900] | 0.845 | [0.823, 0.866] | 0.863 | [0.844, 0.882] |
| Hong Kong | 0.866 | [0.851, 0.880] | 0.813 | [0.792, 0.833] | 0.847 | [0.830, 0.863] |
| Indonesia | 0.885 | [0.868, 0.901] | 0.810 | [0.783, 0.836] | 0.838 | [0.816, 0.861] |
| India | 0.896 | [0.881, 0.911] | 0.818 | [0.792, 0.843] | 0.870 | [0.852, 0.889] |
| Malaysia | 0.924 | [0.915, 0.934] | 0.872 | [0.856, 0.887] | 0.889 | [0.876, 0.902] |
| Spain | 0.928 | [0.921, 0.934] | 0.880 | [0.868, 0.891] | 0.886 | [0.874, 0.895] |
| Taiwan | 0.938 | [0.932, 0.944] | 0.921 | [0.913, 0.928] | 0.910 | [0.902, 0.919] |
| Thailand | 0.873 | [0.854, 0.892] | 0.809 | [0.781, 0.838] | 0.848 | [0.825, 0.870] |
| Vietnam | 0.885 | [0.871, 0.899] | 0.822 | [0.800, 0.844] | 0.860 | [0.843, 0.877] |

Note: 95% CI = 95% Confidence Interval.

Table S5. Frequency of cross-loadings ≥ 0.30 across ten regions

| Item | Target factor | Number of regions with cross-loading ≥ 0.30 on another factor | Frequency | Most affected non-target factor(s)(Regions) |
| --- | --- | --- | --- | --- |
| 3 | Depression | 6 | 60% | Anxiety (CHN, GHA, TWN), Stress (IND, MYS, VNM) |
| 5 | Depression | 9 | 90% | Anxiety (CHN, GHA, TWN), Stress ( CHN,CHA, HKG,IDN,IND, MYS,TWN, THA,VNM) |
| 13 | Depression | 9 | 90% | Stress (CHN,HKG,IDN,IND, MYS,TWN,ESP, THA,VNM) |
| 9 | Anxiety | 8 | 80% | Depression (GHA), Stress (CHN,GHA,HKG,IDN,MYS,TWN,ESP,THA) |
| 15 | Anxiety | 8 | 80% | Depression (CHN, GHA,HKG, ESP, TWN), Stress (GHA,IDN,MYS,THA) |

**Note:** CHN (Mainland China) GHA (Ghana) HKG (Hong Kong) IDN (Indonesia) IND (India) MYS (Malaysia) ESP (Spain) TWN (Taiwan) THA (Thailand) VNM (Vietnam)

Table S6. Comparison of Inter-Factor Correlations in DASS-21: Oblique Three-Factor Model vs. Three-Factor Exploratory Structural Equation Model (ESEM) Model

| Region | Oblique three-factor model | | | Three-factor ESEM model | | |
| --- | --- | --- | --- | --- | --- | --- |
|  | Dep-Anx | Dep-Stress | Anx-Stress | Dep-Anx | Dep-Stress | Anx-Stress |
| Mainland China (*n*=6278) | 0.95 | 0.93 | 0.97 | 0.55 | 0.50 | 0.60 |
| Ghana (*n*=466) | 0.94 | 0.97 | 0.99 | 0.60 | 0.55 | 0.55 |
| Hong Kong (*n*=759) | 0.88 | 0.89 | 0.93 | 0.47 | 0.49 | 0.49 |
| Indonesia (*n*=458) | 0.82 | 0.82 | 0.96 | 0.44 | 0.47 | 0.47 |
| India (*n*=454) | 0.85 | 0.88 | 0.98 | 0.59 | 0.58 | 0.46 |
| Malaysia (*n*=624) | 0.83 | 0.87 | 0.96 | 0.48 | 0.56 | 0.59 |
| Spain (*n*=1024) | 0.83 | 0.83 | 0.89 | 0.53 | 0.56 | 0.62 |
| Taiwan (*n*= 968) | 0.97 | 0.95 | 0.95 | 0.66 | 0.59 | 0.62 |
| Thailand (*n*=418) | 0.84 | 0.86 | 0.94 | 0.47 | 0.49 | 0.49 |
| Vietnam (*n*=610) | 0.78 | 0.90 | 0.88 | 0.58 | 0.48 | 0.58 |

Dep-Anx = The association between depression and anxiety; Dep-Stress = The association between depression and stress; Anx-Stress = The association between anxiety and stress.

Table S7. Fit Indices for Language Measurement Invariance in Three-Factor ESEM

| **Multi-Group Comparison Across Language Groups** | | | | | | | | | | | |
| --- | --- | --- | --- | --- | --- | --- | --- | --- | --- | --- | --- |
| Model | χ^2^ | df | Model comparison | Δχ^2^ | Δdf | CFI | ΔCFI | RMSEA | ΔRMSEA | SRMR | ΔSRMR |
| M0 | 3255.15 | 1050 |  |  |  | 0.991 |  | 0.047 |  | 0.025 |  |
| M1 | 5128.23 | 1374 | M1–M0 | 1873.08 | 324 | 0.984 | -0.007 | 0.053 | 0.006 | 0.044 | 0.019 |
| M2 | 9487.81 | 1482 | M2–M1 | 4359.81 | 108 | 0.966 | -0.018 | 0.075 | 0.022 | 0.059 | 0.015 |

**Note:** CFI, comparative fit index; RMSEA, root mean square error of approximation; SRMR, standardized root mean square residual; M0, baseline model; M1, a factor-loading constrained model; M2, a factor-loading and item-intercept constrained.
