# Supplementary material for: A cross-sectional study examining the reliability and invariance of the Depression, Anxiety, and Stress Scale–21 (DASS-21) across ten countries/regions
Source: PeerJ. 2026 Sep 29;14:e21745. doi: 10.7717/peerj.21745 (PMC13632677; doi:10.7717/peerj.21745)
Supplement: Supplementary file 2 [file peerj-14-21745-s003.docx]

STROBE Statement—checklist of items that should be included in reports of observational studies

|  | Item No. | Recommendation | Page  No. | Relevant text from manuscript |
| --- | --- | --- | --- | --- |
| **Title and abstract** | 1 | (*a*) Indicate the study’s design with a commonly used term in the title or the abstract | 1-2 | cross-sectional study |
|  |  | (*b*) Provide in the abstract an informative and balanced summary of what was done and what was found | 2 | This study evaluated the factor structure of DASS-21 by comparing five models: unidimensional, oblique three-factor, bifactor, three-factor ESEM, and bifactor ESEM. For the best-fitting model, internal consistency, discriminant validity, and measurement invariance across regional and demographic groups were examined. The three-factor ESEM model showed the best fit with satisfactory internal consistency, acceptable discriminant validity and convergent validity. Measurement invariance testing supported configural and partial metric invariance across regional clusters (East Asian, Southeast Asian, and Others), but scalar invariance was not achieved, limiting direct cross-regional comparisons. In contrast, scalar invariance was established across age, gender, data collection code (online versus offline), and pandemic timing (onset versus post-COVID-19). |
| Introduction | | | |  |
| Background/rationale | 2 | Explain the scientific background and rationale for the investigation being reported | 2-5 | High prevalence rates of depression, anxiety and stress among university students needs the reliable and valid screening tool. The competing factor structure and the measurement invariance of DASS-21made this investigation. |
| Objectives | 3 | State specific objectives, including any prespecified hypotheses | 5 | First, determine which factor structure best represents the DASS-21 across culturally diverse university populations by comparing five competing models. Second, examine whether the optimal model demonstrates adequate psychometric properties. Third, test the measurement invariance across ten regions involved in this study. |
| Methods | | | |  |
| Study design | 4 | Present key elements of study design early in the paper | 6 | secondary data collected between 2021 and 2023 as part of a large international mental health project led by the present research team |
| Setting | 5 | Describe the setting, locations, and relevant dates, including periods of recruitment, exposure, follow-up, and data collection | 6 | Data collection was aligned across ten regions: across ten regions: Mainland China, Ghana, Hong Kong, India, Indonesia, Malaysia, Spain, Taiwan, Thailand, and Vietnam, and conducted between June 2021 and October 2023 (See Table S1 for detail) |
| Participants | 6 | (*a*) *Cohort study*—Give the eligibility criteria, and the sources and methods of selection of participants. Describe methods of follow-up  *Case-control study*—Give the eligibility criteria, and the sources and methods of case ascertainment and control selection. Give the rationale for the choice of cases and controls  *Cross-sectional study*—Give the eligibility criteria, and the sources and methods of selection of participants |  |  |
|  |  | (*b*) *Cohort study*—For matched studies, give matching criteria and number of exposed and unexposed  *Case-control study*—For matched studies, give matching criteria and the number of controls per case | 6 | Matching criteria: (i) current enrollment as a university student and (ii) absence of previously diagnosed mental health disorders. |
| Variables | 7 | Clearly define all outcomes, exposures, predictors, potential confounders, and effect modifiers. Give diagnostic criteria, if applicable | 6-7 | Outcomes: factor structure of DASS-21, and the reliability, validity of the optimal structure, measurement invariance across ten regions |
| Data sources/ measurement | 8* | For each variable of interest, give sources of data and details of methods of assessment (measurement). Describe comparability of assessment methods if there is more than one group | *6* | *Participants were university students recruited through convenience sampling, with support from student affairs offices at each institution. Data collection methods varied depending on local infrastructure and conditions—some regions used country-specific online platforms, while others employed paper-and-pencil formats. Detailed information on data collection modes and study locations is provided in Table S1.* |
| Bias | 9 | Describe any efforts to address potential sources of bias | 6 | minimize temporal bias, conduct a power analysis |
| Study size | 10 | Explain how the study size was arrived at | 6-7 | Conduct a power analysis with the *semPower* package in R and find that over 400 participants across all regions, ensure robust and stable parameter estimation. |

Continued on next page

| Quantitative variables | 11 | Explain how quantitative variables were handled in the analyses. If applicable, describe which groupings were chosen and why | - | - |
| --- | --- | --- | --- | --- |
| Statistical methods | 12 | (*a*) Describe all statistical methods, including those used to control for confounding | 7 | ESEM, two-stage multi-group CFA |
|  |  | (*b*) Describe any methods used to examine subgroups and interactions | - | - |
|  |  | (*c*) Explain how missing data were addressed | 8 | two-stage robust maximum likelihood approach |
|  |  | (*d*) *Cohort study*—If applicable, explain how loss to follow-up was addressed  *Case-control study*—If applicable, explain how matching of cases and controls was addressed  *Cross-sectional study*—If applicable, describe analytical methods taking account of sampling strategy | - | - |
|  |  | (*e*) Describe any sensitivity analyses | 8 | To validate the robustness of findings, sensitivity analyses were conducted using a two-stage robust maximum likelihood approach [58] to handle missing data. These results were compared with those obtained using FIML estimation. |
| Results | | | | |
| Participants | 13* | (a) Report numbers of individuals at each stage of study—eg numbers potentially eligible, examined for eligibility, confirmed eligible, included in the study, completing follow-up, and analysed | *8* | *Table S1: Mainland China: 6278; Chana: 466; HongKong: 759, Indonesia: 458, India:454, Malaysia: 624, Spain:1024; Taiwan: 973, Thailand:413, Vietnam: 610* |
|  |  | (b) Give reasons for non-participation at each stage | - | - |
|  |  | (c) Consider use of a flow diagram | 8 | Figure 1 |
| Descriptive data | 14* | (a) Give characteristics of study participants (eg demographic, clinical, social) and information on exposures and potential confounders | *8* | *Table S1:Information of Participants.* |
|  |  | (b) Indicate number of participants with missing data for each variable of interest | *8* | *Missing data were minimal, affecting only a few items: Item 11 (n = 1), Item 12 (n = 2), Item 14 (n = 2), Item 18 (n = 3), and age (n = 5).* |
|  |  | (c) *Cohort study*—Summarise follow-up time (eg, average and total amount) |  |  |
| Outcome data | 15* | *Cohort study*—Report numbers of outcome events or summary measures over time |  |  |
|  |  | *Case-control study—*Report numbers in each exposure category, or summary measures of exposure |  |  |
|  |  | *Cross-sectional study—*Report numbers of outcome events or summary measures | *9-10* | *3 outcome events. The three-factor ESEM model provided the best fit (χ²(df) = 2904.12(150), CFI = 0.964, RMSEA = 0.039) . McDonald's ω coefficients >0.70 for both the entire sample and each individual regions. Measurements results revealed that scalar invariance was not achieved in any of the three regional groups. While Groups 1 and 3 met criteria for metric invariance (i.e., equal factor loadings), Group 2 did not. Scalar invariance (equal intercepts) was unsupported across all groups, with intercept constraints significantly degrading model fit beyond Chen's criteria [57]. Scalar invariance was supported across all these subgroups:age (≤20 vs. ≥21 years), gender (men vs. women), data collection mode (online vs. offline), and pandemic timing (onset vs. post-COVID-19).* |
| Main results | 16 | (*a*) Give unadjusted estimates and, if applicable, confounder-adjusted estimates and their precision (eg, 95% confidence interval). Make clear which confounders were adjusted for and why they were included | 10 | Tables 1-6 |
|  |  | (*b*) Report category boundaries when continuous variables were categorized | - | - |
|  |  | (*c*) If relevant, consider translating estimates of relative risk into absolute risk for a meaningful time period | - | - |

Continued on next page

| Other analyses | 17 | Report other analyses done—eg analyses of subgroups and interactions, and sensitivity analyses | 10 | Sensitivity analysis |
| --- | --- | --- | --- | --- |
| Discussion | | | | |
| Key results | 18 | Summarise key results with reference to study objectives | 11-13 | Structural validity findings: From traditional CFA to ESEM  Cross-cultural measurement invariance |
| Limitations | 19 | Discuss limitations of the study, taking into account sources of potential bias or imprecision. Discuss both direction and magnitude of any potential bias | 13 | Strengths and Limitions |
| Interpretation | 20 | Give a cautious overall interpretation of results considering objectives, limitations, multiplicity of analyses, results from similar studies, and other relevant evidence | 11-14 | Structural validity findings: From traditional CFA to ESEM  Cross-cultural measurement invariance |
| Generalisability | 21 | Discuss the generalisability (external validity) of the study results | 13 | The sample was geographically imbalanced, with eight regions from Asia and limited representation from Europe and Africa, and none from the Americas or Oceania—limiting global generalizability. |
| Other information | |  | | |
| Funding | 22 | Give the source of funding and the role of the funders for the present study and, if applicable, for the original study on which the present article is based | Submission system | This work was supported by a special grant from the Taishan Scholars Project [Project No. tsqn202211130]. |

*Give information separately for cases and controls in case-control studies and, if applicable, for exposed and unexposed groups in cohort and cross-sectional studies.

**Note:** An Explanation and Elaboration article discusses each checklist item and gives methodological background and published examples of transparent reporting. The STROBE checklist is best used in conjunction with this article (freely available on the Web sites of PLoS Medicine at http://www.plosmedicine.org/, Annals of Internal Medicine at http://www.annals.org/, and Epidemiology at http://www.epidem.com/). Information on the STROBE Initiative is available at www.strobe-statement.org.
